# Enteral Tube Nutrition in Anorexia Nervosa and Atypical Anorexia Nervosa and Outcomes: A Systematic Scoping Review

**DOI:** 10.3390/nu17030425

**Published:** 2025-01-24

**Authors:** Namrata Dhopatkar, Johanna L. Keeler, Davide Gravina, Jacinda Gower, Hiba Mutwalli, Sevgi Bektas, Sarah J. Fuller, Hubertus Himmerich, Janet Treasure

**Affiliations:** 1 South London and Maudsley NHS Foundation Trust, London SE5 8AZ, UK; hubertus.himmerich@kcl.ac.uk (H.H.); 2 Centre for Research in Eating and Weight Disorders, Institute of Psychiatry, Psychology and Neuroscience, King’s College, London SE5 8AF, UK; johanna.keeler@kcl.ac.uk (J.L.K.); davide.gravina@kcl.ac.uk (D.G.); hiba.mutwalli@kcl.ac.uk (H.M.); sevgi.bektas1@kcl.ac.uk (S.B.); 3 Department of Clinical and Experimental Medicine, University of Pisa, 56127 Pisa, Italy; 4 South West London and St George’s Mental Health NHS Trust, London SW17 0YF, UK; jacinda.gower@swlstg.nhs.uk; 5 Department of Clinical Nutrition, College of Applied Medical Sciences, Imam Abdulrahman Bin Faisal University, Dammam 31441, Saudi Arabia; 6 Department of Psychology, Hacettepe University, Ankara 06800, Türkiye; 7 Northamptonshire Healthcare NHS Foundation Trust, Northampton NN15 7PW, UK; sarah.fuller@nhs.net; 8 Department of Brain Sciences, Imperial College London, London SW7 2AZ, UK

**Keywords:** enteral tube nutrition, nasogastric, transition, weaning, anorexia nervosa, atypical anorexia nervosa

## Abstract

**Background**: Anorexia nervosa and atypical anorexia nervosa require refeeding as a core part of their treatment, and enteral tube nutrition (ETN) may be needed in some individuals either to supplement or replace oral nutrition. This scoping review aimed to explore outcomes associated with phases of ETN, including initial nutrition, transition from enteral nutrition to oral intake, and to overall nutrition; **Methods**: The Preferred Reporting Items for Systematic Reviews and Meta-Analyses extension for scoping reviews checklist was used. A systematic search was performed using the Ovid and the Web of Science databases, using relevant search terms identifying 37 studies to be included in the review. Quantitative and qualitative data were synthesised and presented; **Results**: ETN resulted in similar or higher weight gain than oral nutrition. Refeeding syndrome parameters were comparable between ETN and oral nutrition with some indication that prophylactic phosphate supported mitigation of hypophosphataemia. Global psychological parameters related to the eating disorder improved with nutrition; however, there was an indication that weight and shape concerns did not improve during this period. There was a lack of evidence related to transition from ETN to oral intake. Qualitative data indicated meanings attached to the tube, suggesting that additional support may be needed for the transition away from the tube. Furthermore, consideration may be required to support individuals in mitigating trauma related to ETN under restraint; **Conclusions**: ETN, when required, is a viable alternative to oral intake. Results indicated the need for further research, especially in the transition from ETN to oral intake with regard to strategies of transition and support, and outcomes related to these strategies.

## 1. Introduction

Anorexia nervosa (AN) is an eating disorder characterised by restriction of oral nutrition, resulting in a significantly low weight, along with an associated fear of weight gain and body dysmorphia [1]. It is known to be correlated with significant morbidity and has one of the highest mortality rates among mental health disorders with a standardised mortality ratio of ~5 [2]. Atypical anorexia nervosa (AAN), while characterised by body image fears like AN, does not meet the criteria for significantly low weight. However, AAN is associated with significant weight loss and, like AN, is related to significant medical instability [3]. Nutritional rehabilitation, to reduce risk and promote health, is key in the treatment of AN and AAN, typically through oral nutritional support [4], which would include regular food intake, and oral nutritional supplement (ONS) drinks if needed. However, as food restriction is part of both illnesses, it may be difficult for sufferers to accept oral nutrition; therefore, nutrition via other means, such as enteral tube nutrition (ETN), may be required. ETN may also be used in mitigating the risks of medical instability, especially while reintroducing nutrition, and may be used for those deemed most at risk [5] or as part of a protocol for those needing to be admitted for refeeding [6]. In situations where ETN is refused, there may be a need for its compulsory administration as a way of reducing the risk to life, with the use of appropriate legal means [5].

When required in AN or AAN, ETN is typically administered via a nasogastric (NG) tube that delivers liquid nutrition directly into the stomach. However, other tubes may be used occasionally; for instance, the percutaneous endoscopic gastrostomy (PEG) tube—a more permanently placed tube delivering nutrition to the stomach. NG tubes have the disadvantage of a higher likelihood of displacement, especially with tube tampering (for example, an individual attempting tube removal) or with vomiting. Additionally, the use of a NG tube may encourage the competitive element of an eating disorder as a ‘visual’ symbol of its severity and of the individual’s alliance with the eating disorder. A disadvantage of a PEG tube is that it requires an endoscopic procedure with placement across the stomach wall, making it more invasive. Nutrition, typically polymeric formulas (containing whole proteins, carbohydrates, lipids, and micronutrients) or ONS drinks, may be administered in both these tubes in similar ways; for example, as a continuous feed administered via an enteral pump over a defined time period or as ‘boluses’ akin to meals or snacks. When indicated, for example, in gastroparesis and with significant compensatory or physiological vomiting [5,7], the feed may be administered via tubes placed into the jejunum, such as nasojejunal tubes and gastrostomy tubes with jejunal extensions.

Refeeding an individual who has been restricting their food intake, with or without compensatory behaviours, be it through oral intake (OI) or ETN, involves a shift in the metabolic status from the ‘starved’ to a ‘fed’ state, thus increasing their risk of refeeding syndrome (RFS) [8]. With the increased availability of nutrients, a combination of increased blood glucose with a corresponding increase in insulin secretion, electrolyte, and fluid shifts between the extra- and intra-cellular compartments of the body, and an increased need for vitamins involved in metabolic processes can be seen. This may result in features such as hypophosphataemia, hypokalaemia, and hypomagnesemia. Clinical presentation of RFS can range from a combination of features such as oedema, cardiac arrythmias, and cardiac failure which can, on occasion, be fatal [8]. Refeeding hypophosphataemia (RH) has often been used as an indicator of RFS, both being linked with the severity of malnutrition [8]. Various methods have been trialled to mitigate the risk of RFS and its morbidity, including a graded reintroduction of nutrition [9], reducing energy provision through carbohydrates in the initial phase of nutrition [10], and the use of continuous ETN [11]. In restrictive eating disorders, such as AN and AAN, the effects of undernutrition pose a significant physical health risk to individuals; thus, the risk of managing these while also mitigating RFS risk needs careful consideration. A recent guidance proposes starting refeeding at least at 10–20 kcal/kg/day (1400 kcal/day for those under 18 years old) in those with a high RFS risk, with a steady increase to 60 kcal/kg/day, and careful monitoring of their physical state, to support safe refeeding [5].

ETN use may be considered inevitable in some cases, such as food refusal and medical severity. However, it is important to explore outcomes associated with ETN, including physical health parameters, potential adverse outcomes, length of stay data, psychological parameters, qualitative data, and long-term outcomes to acquire a broader picture of its effects in AN and AAN.

Resistance to nutrition of any kind, be it oral or ETN, may prolong the time spent in the hospital by service users. Conversely, acceptance of ETN by an individual may increase dependence on medicalised nutrition and impair their capacity to take back responsibility for their own nourishment, potentially prolonging an admission and time away from their community. Thus, exploring the nutritional transition from ETN back to OI is of importance.

The outcomes associated with ETN use, its efficacy, and the risks involved in its administration for eating disorders and AN have been previously reviewed [12,13]. The aim of this systematic scoping review is to add to the evidence base by evaluating ETN outcomes in AN and AAN at various stages, including initial refeeding and transition from ETN to OI, to rationalise transition strategies both initially, while starting nutrition, and when weaning from ETN to OI.

## 2. Methods

This review was conducted based on the Preferred Reporting Items for Systematic Reviews and Meta-Analyses Extension for Scoping Reviews (PRISMA-ScR) checklist [14]. A systematic search was performed utilising the following databases: Embase (Ovid), MEDLINE (Ovid), APA PsycInfo (Ovid), and the Web of Science (WoS) Core Collection from inception until 5th June 2024. A combination of search terms was used, including ‘anorexia nervosa’ or ‘atypical anorexia nervosa’ with ‘enteral nutrition’ or ‘nasogastric’ or ‘tube feeding’ or ‘gastrostomy’ or ‘PEG’ or ‘*jejunal’ or ‘jejunostomy’ or ‘transition’ or ‘weaning’ or ‘refeeding’ or ‘nutrition* support’ or ‘nutrition* supplement’ or ‘meal support’ or ‘meal based’.

For inclusion in the scoping review, studies had to be (a) original research presenting outcomes related to ETN and (b) in participants with a diagnosis of AN or AAN. Exclusion criteria were (a) case reports, case series, and conference abstracts, (b) studies published in languages other than English, (c) animal studies, (d) reviews, letters, comments, books, and editorials, and (e) studies whose outcomes could not be related to ETN or to the diagnosis of AN or AAN. References were exported to and de-duplicated in EndNote 20 and Rayyan [15].

The initial screening of titles and abstracts for the inclusion and exclusion criteria was undertaken by two researchers independently (ND and DG). Full texts of possible articles were examined, and any difference in opinion was resolved through discussion with supervising authors (JT and HH) and other co-authors. References from relevant articles were also screened to include all pertinent studies. The protocol for the review was registered prospectively on the Open Science Framework (https://doi.org/10.31219/osf.io/2v6jf). While the registered protocol did not specify the search terms ‘*jejunal’, ‘jejunostomy’, ‘PEG’ or ‘gastrostomy’, it was thought to be important to amend the search by adding these terms of possible routes of ETN administration within these eating disorders for the thoroughness of the search.

Data from quantitative and qualitative studies identified as eligible were extracted (ND) and a third of these data were independently verified (JG). The quality of the studies was examined using the Joanna Briggs Institute Critical Appraisal Tools [16,17]. However, as this was a scoping review, all eligible articles were included to explore the breadth of available research. Data extraction was followed by synthesis of information guided by the objectives of the study and emerging categories based on methodology and outcomes studied.

## 3. Results

**Figure 1:**
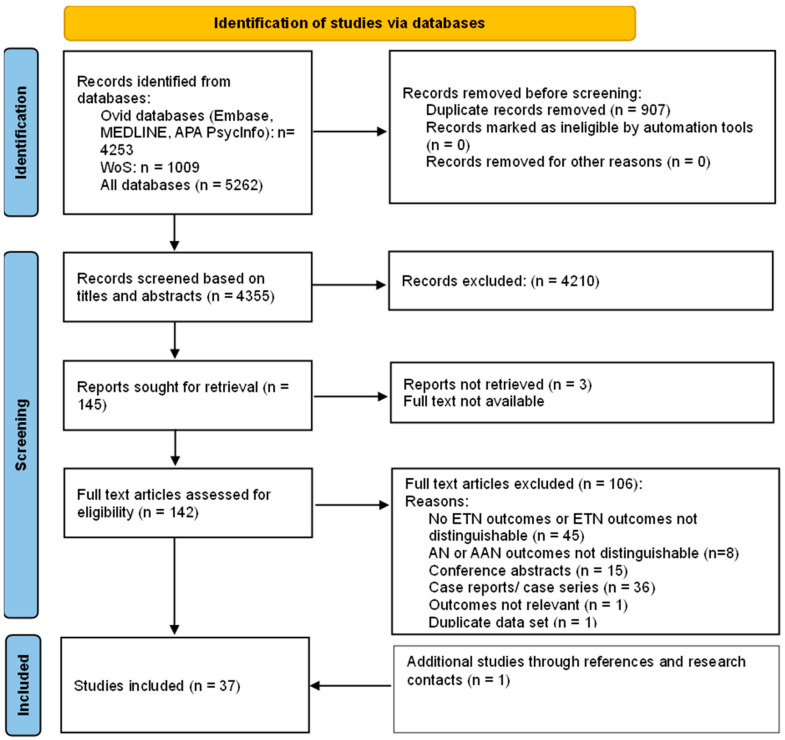
PRISMA flow diagram illustrating the process for study selection.

Figure 1 illustrates the flowchart of the search methodology to identify eligible studies as per PRISMA guidelines [14]. The original database search yielded 5262 results, and after removing duplicates, 4355 references remained. Titles and abstracts were screened for eligibility, of which 145 articles were deemed eligible for the full-text examination, which yielded 36 eligible studies. One additional study was included from contact with research authors. Thus, 37 studies were identified as eligible for inclusion in the scoping review.

### 3.1. Baseline Data and Study Designs

#### 3.1.1. Study and Participant Characteristics

Study and participant characteristics, as well as baseline data from the 37 studies, are presented in Table 1. Study locations included Italy (*n* = 10), Australia (*n* = 8), France (*n* = 8), the USA (*n* = 4), Germany (*n* = 2), the UK (*n* = 2), Canada (*n* = 1), Denmark (*n* =1), and Norway (*n* = 1). Of the 31 quantitative studies, 27 were naturalistic cohorts examining practice within their setting, either as single cohorts with an ETN intervention or multiple cohorts with at least one arm examining an ETN intervention. Four studies followed a randomised controlled trial (RCT) design, of which two compared an ETN arm with an OI arm [18,19], while the other two compared two ETN arms [10,20]. All the quantitative studies except one [19] were conducted in hospital settings. Six studies were of a qualitative design, all of which explored participants’ experience of ETN or involuntary treatment with ETN in a hospital setting. Additionally, three studied the views of staff administering ETN [21,22,23], and two explored carers’ views about ETN in their loved ones [22,23].

Of the included studies, 12 were conducted in participants younger than 18 years of age. The combined mean age (SD) of participants (*n* = 1155) was 14.78 (1.64) years in 10 of these studies. A total of 10 studies were undertaken on those above the age of 18 years. In four of these studies, the combined mean age (SD) of participants (*n* = 533) was found to be 25.73 (7.8) years. A total of 14 studies were conducted with participants spanning these age categories. In 13 of these studies, the combined mean age (SD) was 22.04 (7.35) years in their participants (*n* = 1512).

Studies were in predominantly female populations except for one study examining outcomes in male sufferers [24]. All studies included participants diagnosed with AN and four studies included participants with AAN [6,21,25,26]. However, outcomes related to AN and AAN were not reported separately in these studies.

#### 3.1.2. Indications and Methods of ETN

Table 2, Table 3, Table 4 and Table 5 provide details of nutritional provision and related outcomes: initially (Table 2), overall (Table 3 and Table 4), and during the transition from enteral to oral nutrition in the enteral groups (Table 5), within the quantitative studies. Further details of the initial nutritional protocols and the summary of psychological outcomes are presented in Supplementary Tables S1 and S2, respectively. Methods of ETN administration were heterogeneous. Of the quantitative naturalistic studies, 13 specified administering ETN through continuous feeds, one study reported administration via a bolus method, while 13 did not report a continuous or a bolus method. Three RCTs had ETN arms with a continuous feed [10,18,20], while one did not specify the continuous or bolus method [19]. Of the qualitative studies, two described their participants receiving continuous ETN [21,27]. One study reported a mixture of methods for ETN under restraint [28], two were in participants with lived experience of bolus NG under restraint feeds [22,23] and one did not report on methods of ETN administration [29].

Ten studies [6,10,19,20,30,31,32,33,34,35], including three RCTs [10,19,20], initially relied on ETN exclusively as part of the protocol in their ETN arms, with the addition of OI over time. One RCT [18] had an ETN arm with some OI from the start. Seven studies, as part of the protocol, encouraged some OI from the start with ETN supplementing nutrition [24,36,37,38,39,40,41]. Two studies were comparing a protocol change to ETN supplementing OI with a historical protocol with OI only refeeding [24,36]. Nine studies described oral nutritional rehabilitation as first-line, with ONS or ETN used only when required [25,26,42,43,44,45,46,47,48].

Nocturnal ETN, as a way of supplementing energy intake while managing gastrointestinal and psychological discomfort, was stated in two studies [24,36]. Nocturnal ETN was also used in studies as a method of transition from exclusive ETN to using ETN as a supplement to OI [10,20,30,31]. Daytime ETN was specified in one study to reduce AN-related hyperactivity [18]. The route of ETN was mainly via a NG tube with one study specifying ETN through a PEG tube [37]. None of the studies specified feeding through a jejunal route.

Six studies examined outcomes related to nutrition administered involuntarily under appropriate legislation in some or all participants [21,22,23,28,37,42].

Energy prescriptions varied widely in studies. Five studies based in Europe described conservative initial prescriptions with slow increases to the target prescriptions [26,32,33,34,35]. Seven had standard initial prescriptions in their ETN arms, between 1200 and 1800 kcal/day, and variable increases during the study to the target prescription, and were mainly based in Europe and North America [6,25,36,38,39,47,49]. Four studies with higher energy prescriptions starting above 1800 kcal/day, with increases to at least 2400 kcal/day, were all based in Australia [10,20,30,31].

**Table 1:** Study and participant characteristics and baseline data of included studies.

Author, Year *Country*	Objectives	Group Type (Number)	Participant Characteristics				Baseline Anthropometry		Other Baseline Measures/Comments
		Grouping as defined by original study or by route of nutrition	Diagnosis *(tool),* **subtypes**	Gender (%)	Age in years (M ± SD) *(Med ± IQR/**range**)*	DOI in months (M ± SD) *(Med ± IQR/**range**)*	Weight (kg, M ± SD) **BMI (kg/m^2^, M ± SD)**	% IBW/**EBW**/*mBMI*	(M ± SD) Or *(Med ± IQR/range)*
**Randomised trials**									
Madden et al., 2015b [20] *Australia*	Comparing outcomes in admissions for med. stab (MS) versus weight restoration (WR)	1. MS w/NG (n = 41) 2. WR w/NG (n = 41)	AN diagnosis *(DSM-4)* **AN-R (n = 57)** **AN-BP (n = 25)**	1. Female/male (95/5%) 2. Female/male (95/5%)	1. 14.89 ± 1.36 2. 14.88 ± 1.56	1. 7.39 ± 5.42 2. 7.85 ± 6.89	NR **NR**	**1. 77.28 ± 6.67** **2. 79.25 ± 5.95**	
Parker et al., 2021 [10] *Australia*	Evaluating refeeding outcomes comparing low vs. standard CHO feed	1. Low CHO feed w/NG (n = 15) 2. Standard CHO feed w/NG (n = 11)	AN diagnosis *(DSM-5)* **NR**	Female (100%)	Overall: 17.5 ± 1.1 1. 17.5 ± 1.3 2. 17.5 ± 0.9	NR	1. 43.7 ± 4.7 2. 45.1 ± 2.8 **1. 16.3 ± 1.7** **2. 16.7 ± 0.9**	*1. 77.8 ± 9.1* *2. 79.3 ± 5.2*	PO4: 1.: 1.18 ± 0.19 mmol/L 2.: 1.11 ± 0.13 mmol/L K: 1.: 3.75 ± 0.44 mmol/L 2.: 3.72 ± 0.32 mmol/L Mg: 1.:0.94 ± 0.09 mmol/L 2.: 0.94 ± 0.05 mmol/L
Rigaud et al., 2007 [18] *France*	Evaluating outcomes comparing NG and oral refeeding	1. NG (n = 41) 2. OI (n = 40)	AN diagnosis *(DSM = 4)* **AN-R (n = 56)** **AN-BP (n = 25)**	1. Female/male (97/3%) 2. Female/male (98/2%)	1. 22.5 ± 4.5 2. 24.2 ± 3.8	*1. 54 ± 22.8* *2. 38.4 ± 24*	1. 34.0 ± 3.9 2. 34.7 ± 4.3 **1. 12.1 ± 1.5** **2. 12.8 ± 2.0**	NR	-
Rigaud et al., 2011b [19] *France*	Comparing binge-purge symptoms in nut rehab with NG nutrition and CBT vs. CBT with OI in BN and AN-BP	1. NG + CBT (n = 52; n AN = 19) 2. OI + CBT (n = 51, n AN = 17)	AN or BN diagnosis *(DSM-IV)* **AN-BP: (n = 36)**	Female (100%)	1. 27.4 ± 8.1 2. 27.9 ± 6.2	1. 117.6 ± 74.4 1. 100.8 ± 64.8	NR **In AN subgroups:** **1. 15.8 ± 1.3** **2. 16.2 ± 1.0**	NR	-
**Retrospective cohort studies**									
Agostino et al., 2013 [6] *Canada*	Comparing NG versus oral protocols for refeeding	1. NG (n = 31) 2. OI (n = 134)	AN or EDNOS -restrictive form *(DSM IV)* **NR**	1. Female/male (94/6%) 2. Female/male (96/4%)	1. 14.9 ± 2.1 2. 14.9 ± 1.7	NR	NR **1. 16.6 ± 2.2** **2. 16.7 ± 2.3**	1. 82 ± 10 2. 85 ± 13	-
Blikshavn et al., 2020 [42] *Norway*	Evaluating outcomes related to physical restraint in hospital including NG under restraint (NG-R)	1. NG-R (n = 8) 2. No NG-R (n = 30)	AN diagnosis *(DSM-5)* **NR**	Female (89.5%) Male (10.5%)	15.9 ± 1.9	NR	NR **15.2 ± 1.9**	NR	2. No NG-R—study did not specify whether any of this group had NG without restraint
Braude et al., 2020 [46] *Australia*	Evaluating outcomes of a medical stabilisation protocol	1. NG (n = 27) 2. OI (n = 68)	AN diagnosis *(NR)* **AN-R (n = 61)** **AN-BP (n = 34)**	Female (89.5%) Male (10.5%)	*21 (**18–29**)*	NR	NR **17.1 ± 3.8**	-	-
Bufano et al., 1990 [49] *Italy*	Evaluating outcomes of NG refeeding.	1. NG (n = 9)	AN diagnosis *(DSM III)* **AN-R (n = 9)**	Female (100%)	NR	17.0 ± 14.0	34.51 ± 4.48 **NR**	NR	-
Gentile, 2012 [38] *Italy*	Evaluating outcomes of NG refeeding in BMI ≤ 12	1. NG (n = 10)	AN diagnosis *(DSM-IV-TR)* **NR**	NR	23.9 ± 11.1	56.3 ± 47.7	27.9 ± 3.3 **11.2 ± 0.7**	NR	PO4 ↓- n = 3 (30%)
Gentile et al., 2008 [47] *Italy*	Evaluating outcomes of a refeeding protocol including NG when needed in BMI ≤ 13.5	1. NG (n = 32) 2. OI (n = 67) n = 75 completed study overall, n = 32 (100%) from NG gp, n = 43 (64%) from OI gp	AN diagnosis *(DSM-IV-TR)* **NR**	Female (96%) Male (4%)	1. 21.8 ± 9.1 2. 18.9 ± 6.2	1. 45.6 ± 40.8 2. 36.5 ± 42.0	1. 31.9 ± 4.8 2. 32.7 ± 5.2 **1. 12.3 ± 0.9** **2. 12.8 ± 0.7**	NR	Baseline data from ‘completers’ of study
Hanachi et al., 2013 [33] *France*	Evaluation of liver function with refeeding including NG	1. ↑ AST/ALT gp w/ETN (n = 54) 2. ↔ AST/ALT gp w/ETN (n = 72)	AN diagnosis *(DSM-IV-TR and 5)* **AN-R (n = 73)** **AN-BP (n = 53)**	Female (93%) Male (7%)	1. 28.0 ± 9.0 2. 32.0 ± 12.0 *	NR	1. 31.5 ± 6.0 2. 33.1 ± 5.0 **1. 11.2 ± 1.7** 2. 12.7 ± 1.7 *	-	ALT 1.: 174 ± (57–2614) 2.: 49 ± (9–119) * AST: 1.: 133 ± (46–2120) 2.: 27 ± (6–57) * GGT: 1.: 150 ± (17–555) 2. 45 ± (8–233) * ALP: 1.: 128 ± (41–440) 2.: 61 ± (22–122) *
Kells et al., 2022 [25] *USA*	Evaluating factors contributing to RH	1. NG (n = 44) 2. OI (n = 256)	AN or AAN diagnosis *(DSM IV-TR or 5)* **AN-R (n = 255)** **AN-BP (n = 35)** **AAN (n = 6)** **Missing (n = 4)**	Female (88.3%) Male (11.7%)	15.5 ± 2.5	NR	42.8 ± 9.6 **16.3 ± 2.6**	*82 ± 12.1*	
Marchili et al., 2023 [43] *Italy*	Comparing NG nutrition outcomes including those related to the timing of starting NG	1. NG (n = 101) 2. OI (n = 214)	AN diagnosis *(DSM-5)* **NR**	1. Female/male (91/9%) 2. Female/male (88/12%)	Overall: 14.4 ± 1.2 1. 14.6 ± 1.8 2. 14.4 ± 2.3	NR	NR **1. 14.5 ± 1.9** 2. 16.1 ± 2.8 *	NR	BMI percentile, *median (IQR)*: 1. *0.2 (5.1)* 2. 1.9 (19.5) *
Martini et al. 2024 [44] *Italy*	Evaluation of BMI and treatment satisfaction comparing NG and OI	1. NG: (n = 97) 2. OI: (n = 97)	AN diagnosis (DSM 5) **AN-R: (n = 124)** **AN-BP (n = 70)**	Female (100%)	1. NR 2. NR **Overall >18 and <65**	1. 84 ± 108 2. 84 ± 96	1. 37 ± 6 2. 37 ± 5.5 **1. 13.99 ± 1.76** **2. 14.01 ± 1.89**	-	NG and OI group were matched for age, duration of illness, AN subtype, BMI, psychiatric co-morbidities, energy intake, EDE-Q total score and number of hospitalisations via propensity score matching
Nehring et al., 2014 [50] *Germany*	Evaluations of outcomes including longer-term outcomes related to NG nutrition	1. NG (n = 71) 2. OI (n = 137)	AN diagnosis *(ICD 10)* **NR**	Female (100%)	1. 14.3 ± 1.6 2. 15.3 ± 1.6 *	1. 7.9 ± 5.3 2. 12.3 ± 11.3	NR **1. 14.3 ± 1.3** 2. 15.1 ± 1.4 *	NR	1.: NG during any of their admissions 1. vs. 2. BMI percentiles and BMI s.d scores were comparable
Pruccoli et al., 2021 [51] *Italy*	Evaluating outcomes related to the timing of starting NG and atypical antipsychotics (AAP)	1. Early AAP+ early NG (n = 18) 2. Early AAP + late NG (n = 12) 3. Late AAP + early NG (n = 20) 4. Late AAP + late NG (n = 18) 5. NG only (n = 11)	AN diagnosis *(DSM-5)* **AN-R (n = 78)** **AN-BP (n = 1)**	Female (98.7%) Male (1.3%)	Overall:14.6 ± 2.0 1. 14.7 ± 2.3 2. 14.5 ± 1.8 3. 14.3 ± 1.7 4. 15.2 ± 1.5 5. 14.2 ± 2.0	Overall: 14.8 ± 11.5 1. 15.8 ± 14.3 2. 10.3 ± 6.3 3.12.7 ± 9.3 4. 19.0 ± 13.0 5. 15.0 ± 10.3	NR **Overall: 13.7 ± 1.7** **1. 14.2 ± 1.9** **2. 13.5 ± 1.6** **3.13.3 ± 1.6** **4. 13.6 ± 1.2** **5. 13.8 ± 1.6**	NR	-
Pruccoli et al., 2022 [52] *Italy*	Comparing ‘treatment resistance’ (TR-AN) and ‘good outcome’ (GO-AN) in AN and associations.	1. NG (n = 33) 2. OI (n = 43) Both NG and OI methods used in the TR AN (n = 30) and GO AN (n = 46)	AN diagnosis *(DSM-5)* **AN-R (n = 70)** **AN-BP (n = 6)**	Female (94.7%) Male (5.3%)	14.9 ± 1.9	Overall: 14.2 ± 11 TR AN: 18 ± 14.2 GO AN: 11.7 ± 7.4	NR **Overall: 14.3 ± 1.7**	NR	EDI 3-EDRC score sig higher in TR-AN than GO-AN
Pruccoli et al., 2024 [26] *Italy*	Evaluating effect of the use of olanzapine on refeeding syndrome (RFS) features	1. NG (n = 44) 2. Other: OI or PN (n = 69) Overall group separated into RFS (n = 46) and no-RFS (n = 67)	AN or AAN diagnosis (DSM-5) **AN-R (n = 103)** **AN-BP (n = 8)** **AAN (n = 2)**	Female (90.3%) Male (9.7%)	*Overall: 15 (3)*	RFS gp: *11 (9)* No RFS gp: *9 (11)*	**RFS gp: *14 (2)*** **No RFS gp: *14 (3)***	*RFS gp: 71 ± 10* *No RFS gp: 72 ± 10*	2. PN n = 1 in RFS gp. OI numbers not specified.
Rigaud et al., 2010 [40] *France*	Evaluating the use of low sodium versus standard sodium OI during refeeding	1. Low sodium OI w/NG (n = 176) 2. Standard sodium OI w/NG (n = 42) Both groups—similar NG	AN diagnosis *(DSM-4)* **AN-R (n = 116)** **AN-BP (n = 102)**	Female (98%) Male (2%)	1. 23.3 ± 5.1 2. 22.1 ± 4.2	NR	1. 36 ± 3.8 2. 36.6 ± 4.3 **1. 13.2 ± 1.2** **2. 13.8 ± 1.7**	-	1. vs. 2.: FFM and FM n.s
Robb et al., 2002 [36] *USA*	Comparing short-term outcomes in NG vs. OI refeeding in female adolescents with AN	1. NG (n = 52) 2. OI (n = 48)	AN diagnosis *(DSM-4)* **NR**	Female (100%)	1. 14.8 ± 1.9 2. 15.0 ± 1.8	NR	1. 41.1 ± 4.7 2. 42.5 ± 7.6 **1. 15.5 ± 1.7** **2. 16.0 ± 1.8**	NR	1. (n = 52/52) + 2. (n = 28/48)—treated in an adolescent. medical unit until medically stable, then psychiatric unit. 2. n = 20/48—treated in the adolescent medical unit with psychiatric. consultation
Silber et al., 2004 [24] *USA*	Comparing outcomes in NG vs. OI refeeding in male adolescents with AN	1. NG (n = 6) 2. OI (n = 8)	AN diagnosis *(DSM-4 male applicable criteria)* **NR**	Male (100%)	1. 13.8 ± 2.0 2. 14.9 ± 1.7	NR	1. 42.9 ± 10.7 2. 46.2 ± 11.0 **1. 15.3 ± 1.7** **2. 17.4 ± 2.3**	NR	
Zuercher et al., 2003 [41] *USA*	Comparing outcomes of NG vs. OI refeeding in AN	1. NG (n = 155) 2. OI (n = 226)	AN diagnosis *(DSM-4)* **AN-R (n = 180)** **AN-BP (n = 201)**	Female (100%)	1. 25.7 ± 9.6 2. 25.2 ± 8.4	1. 105.6 ± 102 2. 104.4 ± 93.6	1. 38.0 ± 5.5 2. 42.1 ± 5.4 **1. 14.2 ± 1.7** **2. 15.7 ± 1.7**	-	Overall cohort recommended NG, but the OI gp declined NG.
**Prospective cohort studies**									
Born et al., 2015 [37] *Germany*	Evaluating a compulsory refeeding protocol for AN	1. PEG (n = 57) 2. No PEG (n = 11), w/NG (n = 3) and no NG (n = 8)	AN diagnosis *(NR)* **AN-R (n = 32)** **AN-BP (n = 36)**	Female (95.6%) Male (4.4%)	1. 27.2 ± 8.8 2. 22.9 ± 6.1	1. 118.8 ± 84 2. 87.6 ± 66	NR **1. 12.2 ± 1.4** **2. 12.7 ± 1.3**	-	Most participants under ‘legal guardianship’ during admission
Kezelman et al., 2018 [31] *Australia*	Evaluating anxiety symptoms related to rapid refeeding	1. NG (n = 31)	AN diagnosis *(DSM-5)* **AN-R (n = 24)** **AN-BP (n = 7)**	Female (100%)	16.91 ± 1.1	16.7 ± 21.1	NR **16.31 ± 1.94**	NR	Anxiety disorder comorbidity: n = 20 (64.5%)
Madden et al., 2015a [30] *Australia*	Evaluating outcomes from rapid refeeding in adolescents	1. NG (n = 78)	AN diagnosis *(DSM-4)* **AN-R (n = 53)** **AN-BP (n = 25)**	Female (95%) Male (5%)	14.84 ± 1.46	7.60 ± 6.16	40.99 ± 5.72 **NR**	78.37 ± 6.50	
Minano Garrido et al., 2021 [35] *France*	Evaluating muscle strength and peak expiratory flow (PEF) rate with refeeding in BMI < 13	1. NG (n = 23)	AN diagnosis *(DSM-5)* **AN-R (n = 18)** **AN-BP (n = 5)**	Female (100%)	25.6 ± 6.2	110.4 ± 76.8	NR **11.4 ± 1.3**	-	PEF (L/min): 253.3 ± 60 (N: 418 ± 24.5) Muscle strength Medical Research Council (MRC) score: 37.7 ± 7.7 (N: 60) Axial muscle strength impaired *
Murciano et al., 1994 [39] *France*	Evaluating muscle strength and respiratory and diaphragmatic function with refeeding	1. NG (n = 15)	AN diagnosis *(DSM III revised)* **NR**	Female (100%)	24.9 ± 8.7	NR	37.1 ± 4.7 **13.5 ± 1.1**	63 ± NR	FEV1, % predicted.: 87 ± 17
Paccagnella et al., 2006 [32] *Italy*	Evaluating outcomes in NG refeeding when using specific criteria for commencing NG	1. NG (n = 24)	AN diagnosis *(DSM-IV)* **AN-R (n = 19)** **AN-BP (n = 5)**	Female (100%)	18.5 ± 6.18	NR	33.0 ± NR **12.9 ± NR**	-	Cardiovascular symptoms: n > 60% PO4 and K within range
Rigaud et al., 2011a [48] *France*	Evaluation of long-term outcomes in AN after hospitalisation for refeeding	1. NG (n = 262) 2. OI (n = 222)	AN diagnosis *(DSM-IV)* **AN-R (n = 347)** **Other (n = 137)**	Female (95.4%) Male (4.6%)	22.8 ± 4.4	42 ± 16.8	NR **12.8 ± 1.6**	-	
Rigaud et al., 2012 [34] *France*	Evaluating outcomes of refeeding in AN with BMIs < 11	1. NG (n = 41)	AN diagnosis *(DSM-IV)* **AN-R (n = 35)** **Other (n = 6)**	Female (95%) Male (5%)	28.9 ± 5.4	115.2 ± 40.8	25.9 ± 1.4 **10.1 ± 0.57**	-	Low PO4: 17% Low K: 4%
Trovato et al., 2022 [45] *Italy*	Evaluating which refeeding strategy is associated with better outcomes in AN	1. Food only (n = 66) 2. Food + ONS (n = 63) 3. NG + food: (n = 6) 4. NG + food + ONS (n = 51)	AN diagnosis *(DSM-5)* **NR**	Female (89%) Male (11%)	*14 (**13–16**)*	NR	NR NR	NR	n = 33 (18%)—reasons for admission other than nut. rehab. n = 105 (56.5%)—BMI < 14 kg/m^2^; weight loss > 1 kg/week
**Qualitative studies**									
Fuller et al., 2023 [22] *UK*	Evaluating the impact of NG R for AN treatment	1. Patients (n = 7) 2. Carers (n = 13) 3. Staff (n = 16)	1. AN diagnosis (NR) ** NR	1. Female (100%) 2. Female (85%), male (15%) 3. Female (69%), male (31%)	*1. NR (**19–54)***	*1. NR (**36–≥360)***	-	-	1. Lived experience of NG R 2. Carers whose loved ones experienced NG R. 3. Staff involved in NG R decisions and administration.
Fuller et al., 2024 [23] *UK*	Identifying best practice when NG R is needed	1. Patients (n = 7) 2. Carers (n = 13) 3. Staff (n = 16)	1. AN diagnosis (NR) ** NR	1. Female (100%) 2. Female (85%), male (15%) 3. Female (69%), male (31%)	*1. NR ± **19–54***	*1. NR ± **36–≥360***	-	-	1. Lived experience of NG R 2. Carers whose loved ones experienced NG R 3. Staff involved in NG R decisions and administration
Halse et al., 2005 [29] *Australia*	Evaluating meanings and perceptions attached to NG	1. Overall (n = 23) n = 17/23 discussed NG	AN diagnosis *(NR)* **NR**	Female (100%)	14.8 ± NR	NR	NS **15.6 ± NS**	-	n = 2: first admission n = 14: 2–5 previous admissions
Kezelman et al., 2016 [27] *Australia*	Evaluating experiences of rapid refeeding	1. NG (n = 10)	AN diagnosis *(DSM-5)* **AN-R (n = 9)** **AN-BP (n = 1)**	Female (100%)	17.5 ± 0.97	*NR ± **1–36***	NS **16.12 ± 1.53**	-	n = 7 had comorbid diagnoses (1 or more) of phobias, anxiety disorders, OCD and depression
Mac Donald et al., 2023 [28] *Denmark*	Evaluating the impact of multiple IT events in the context of AN treatment	1. Overall (n = 7). Lived experience of 5 or more IT events including NG R	AN diagnosis (NR) **	Female (100%)	>18	*-*	-	-	n = 4 continued to have AN/AAN. n = 6 current psychiatric co-morbidities.
Matthews Rensch et al., 2023 [21] *Australia*	Acceptability and views of the use of NG nutrition	1. NG (n = 8) 2. Staff (n = 12)	AN, OSFED or AAN diagnosis *(NR)* **AN (n = 6)** **AAN (n = 1)** **OSFED (restrictive subtype) (n = 1)**	1. Female (100%) 2. NR	*1. 22* ***(18–27)***	*1. NR ± **7.5–168***	-	-	n = 4: first admission: n = 2: second admission n = 2: >2 admissions

↑: high; ↔: normal range; ↓: low; AAN: atypical anorexia; ALP: alkaline phosphatase; ALT: alanine transaminase; AN: anorexia nervosa; AN-BP: anorexia nervosa binge-purge subtype; AN-R: anorexia nervosa restrictive subtype; AST: aspartate transferase; BMI: body mass index; CHO: carbohydrate; DSM: Diagnostic and Statistical Manual; DOI: duration of illness; EBW: expected body weight; EDE- Q: eating disorder examination questionnaire; EDI: eating disorder inventory; EDNOS: eating disorder not otherwise specified; EDRC: eating disorder risk composite score; FEV1: forced expiratory volume 1; FFM: fat-free mass; FM: fat mass; GGT: gamma glutamyl transpeptidase; GO-AN: good outcome anorexia nervosa; IBW: ideal body weight; ICD: International Classification of Diseases; IT: involuntary treatment; K: potassium; mBMI: median body mass index; Mg: magnesium; MS: medical stabilisation; NG: at least some nutrition provided through nasogastric tube feeding; NG R: nasogastric tube feeding under restraint; NR: not reported; NS: not specified; n.s: not significantly different; OCD: obsessive–compulsive disorder; OI: oral intake only; ONS: oral nutritional supplements; OSFED: other specific feeding or eating disorder; PEG: percutaneous endoscopic gastrostomy providing at least some nutrition; PEF: peak expiratory flow; PN: parenteral nutrition; PO4: phosphate; TR-AN: treatment-resistant anorexia nervosa; w/NG: with NG nutrition; WR: weight restoration; * significantly different; ** lived experience of AN treatment.

**Table 2:** Summary of initial nutrition and related outcomes.

Author, Year	Cohort (s), Specifics, (n)	Initial Nutritional Overview (Within 2 Weeks of Refeeding Initiation)				Outcomes	Adverse Outcomes
		Initial Prescription kcal/d ^a^ *(Actual Intake)* M ± SD ^a^	Kcal Increase per Day ^a^ (kcal/d ^a^)	Target kcal/Day ^a^ *(Actual Intake)* M ± SD ^a^	Prophylactic PO4 Given	Weight: kg, BMI: kg/m^2^ Weight gain/week: kg M ± SD or *Median (Range)*	M ± SD or *Median (Range)* Occurrence: n, % PO4: Serum Phosphate, K: Serum Potassium, Mg: Serum Magnesium Serum Levels: mmol/L ^a^
Studies with participants < 18 years of age							
Agostino et al., 2013 [6]	1. NG (n = 31)	1500–1800 (1617 ± 276 *)	200	2276 ± 123	Y	1. vs. 2.: - Weight gain week 1: 1.22 ± 1 vs. 0.08 ± 0.9 * - Weight gain of at least 0.5 kg/week: 84% vs. 32% - Patients not gaining weight or losing weight: 6% vs. 51%	1. vs. 2.: - Moderate ↓ PO4: n = 0 vs. n = 3 (1.8%) - ↓ K: n = 1 (3.2%) vs. n = 1 (0.7%)—both h/o laxative abuse - No RFS in either group
	2. OI (n = 134)	1000–1200 (1069 ± 212 *)	150	2373 ± 220	NR		
Madden et al., 2015a [30]	1. NG (n = 78)	2400	As required	Max 3800	Y	Weight gain: - Week 1: 2.79 ± 1.27 - Week 0–0.5 (NG predominant): 1.78 ± 1.78 * - Week 0.5–1 (OI predominant): 1.01 ± 0.81	No ↓ PO4, hypoglycaemia or RFS
Pruccoli et al., 2024 [26]	1. NG (n = 44)	15–20 kcal/kg/d (high risk 5–10)	NR (↑ every 2–3 days)	RFS gp: (*1378 ± 289*) No RFS gp: (*1357 ± 231*)	NR	NR	1. vs. 2.: RFS occurrence: n = 18/44 (41% of NG gp) vs. n = 28/69 (41% of ‘other’ gp) No RFS: n = 26/44 (59% of NG gp) vs. n = 41/69 (59% of ‘Other’ gp) Of ‘RFS’ occurrence gp (n = 46): 39% from NG group, 61% from ‘Other’ gp.
	2. Other (mainly OI) (n = 69)						
Robb et al., 2002 [36]	1. NG (n = 52)	1800 (NG: 600, OI:NG = 2:1)	400 (day 2), 200 (day 3)	Energy for target weight gain: 1–2 kg/week	N	NR	1: - Anti-anxiety medication given for tube placement: n = 2 (3.8%) - Tube removed, needing replacement: n = 3 (6%) - Epistaxis: n = 6 (12%) - Nasal irritation: n = 15 (29%) - No RFS or aspiration pneumonia
	2. OI (n = 48)	NR	NR				
Silber et al., 2004 [24]	1. NG (n = 6)	NR	NR	NR	NR	NR	1: - Nasal irritation: n = 1 (17%) - Epistaxis: n = 1 (17%) - No RFS or aspiration pneumonia
	2. OI (n = 8)						
Trovato et al., 2022 [45]	4 gps: -NG + food (n = 6) -NG + food + ONS (n = 51) -Food only (n = 66) - Food + ONS (n = 63)	40 kcal/kg/d	NR	At least 40 kcal/kg/d (NG: max 1500)	NR	NR	No RFS, or electrolyte abnormalities in any gp
Studies with participants ≥ 18 years of age							
Braude et al., 2020 [46]	1. NG (n = 27)	NR	NR	NR	NR	In the overall cohort: - Weight gain over LOS: 1.4 ± 2.9 - LOS: *9.6 (5.8–19.7) days*	In overall cohort: -Refeeding electrolyte derangement in 26.3% - Hypoglycaemia in 11.1% - NG gp associated with: ↓ K. (OR: 9.4 95% CI: 1.8–50.3) * - NG not associated with ↓ PO4 or ↓ Mg
	2. OI (n = 68)						
Rigaud et al., 2010 [40]	1. Low sodium OI w/NG (n = 176)	NR	NR	Week 1: (*1583 ± 207*)	NR	1. vs. 2.: - Weight gain week 1: 0.73 ± 0.18 vs. 1.42 ± 0.23	Overall cohort: - No NG-related severe adverse effects - No RFS - Sinusitis: n = 12 (6%) 1. vs. 2.: - Oedema: n = 11 (6%) vs. n = 9 (21%) *
	2. Std sodium OI w/NG (n = 42)			Week 1: (*1638 ± 234*)			
Rigaud et al., 2007 [18]	1. NG (n = 41)	NR	NR	Energy for target weight gain 1 kg/week (if BMI < 12.5 kg/m^2^: week 1: <30 kcal/kg/d)	Y	1. vs. 2.: - OI similar - Overall energy intake higher * (as energy via NG + OI in NG gp) - Hunger rating higher * in first 2 weeks. - BP episode complete cessation in week 1: 80% vs. 50% in AN-BP participants * NG gp: AN-BP vs. AN-R: found NG more ‘useful’ in week 1 * AN-R vs. AN-BP: anxiety associated. with OI alongside NG higher *	No RFS, severe ↓ K or ↓ PO4 in either group 1.: - Gastro-oesophageal reflux: n = 2 (5%) - Sinusitis: n = 2 (5%)
	2. OI (n = 40)						
Rigaud et al., 2011b [19]	1. NG + CBT (n = 52) (AN subgp: n = 19)	NR	NR	NR	NR	1. vs. 2.: - BP abstinence at day 8: 79% vs. 16% * - Similar results in AN and BN subgps	NR
	2. OI + CBT (n = 51) (AN subgp n = 17)						
Studies with participants across both age categories							
Gentile, 2012 [38]	1. NG (n = 10)	*Median: 1199*	NR	2508	Y	Baseline vs. day 15: - BMI: 11.2 ± 0.7 vs. 12.9 ± 0.9 - Weight: 27.9 ± 3.3 vs. 32.0 ± 3.8 - Weight gain over 15 days: *4.35 kg*	- PO4, Mg, K within range during refeeding - No RFS - No gastrointestinal symptoms reported by participants - No aspiration or tube malposition
Kells et al., 2022 [25]	1. NG (n = 44)	1000–3000 *(1714 ± 324)*	250	NR	Y	1. vs. 2.: - Initial prescription higher *. - Formula given over admission higher * - Weight gain similar between groups	1. vs. 2.: - ↓ PO4 risk 3x higher - K and Mg nadir n.s.
	2. OI (n = 256)						
Murciano et al., 1994 [39]	1. NG (n = 15)	NR	NR	Week 1: (*1824 ± 399*)	NR	Day 0 vs. Day 7 of refeeding: - Weight: 37.1 ± 4.7 vs. 39.2 ± 4.3 - Diaphragmatic function improvement at day 7 * - no muscle mass increase at day 7 n.s.	NR
Paccagnella et al., 2006 [32]	1. NG gp (n = 24)	<1000	NR	Day 12: 1455	NR	On starting NG: - Abdominal symptoms ↓ - Cardiovascular symptoms ↓	- Mild RFS features: n = 2 (8%)
Parker et al., 2021 [10]	1. Low CHO feed (n = 15)	*1260–1890*	Day 2: 2500	Week 1 median (range): *3350 (3188, 3350)*	Y—if PO4 ≤ 1 mmol/L pre-refeeding	1. vs. 2. - Weight gain: week 1: 2.7 ± 1.9 vs. 2.7 ± 1.6 - Day when med stab reached: *2.0 (0.0,* 3.3) vs. 2.0 *(0.8, 5.0)*	1. vs. 2.: - lower levels of RH in 1. * (day of RH occurrence: 2.9 ± 1.2) - PO4 at week 1: 1.06 ± 0.15 vs. 0.88 ± 0.12 * - RH not correlated to %mBMI - Requirement for K and Mg supplementation during refeeding: n.s - Hypoglycaemia: 0/14 vs. 1/10 n.s. - No RFS, oedema or admission to the intensive care unit reported in either group
	2. Standard CHO feed (n = 11)			Week 1 median (range): *3325 (2844, 3350)*			
Rigaud et al., 2012 [34]	1. NG (n = 41)	25 kcal/kg/d	10 kcal/kg/d (in 2 days)	Day 6–10: 40 kcal/kg/d	Y	Baseline vs. week 1: - Weight: 25.9 ± 1.4 vs. 27.1 ± 1.0 - BMI: 10.1 ± 0.28 vs. 10.7 ± 0.2 - FFM: 24.1 ± 1.1 vs. 25.3 ± 0.9 - FM: 1.6 ± 0.9 vs. 1.8 ± 0.7 Baseline neuropathy in n = 6 (15%) → improved within 3 weeks of refeeding.	↓ PO4: n = 4 (10%) Refeeding oedema: n = 9 (22%)
Zuercher et al., 2003 [41]	1. NG (n = 155)	NR	300 every 3 days	Energy for the target weight gain of >1 kg/week	NR	NR	1. vs. 2.: - Oedema: 19% vs. 13% -gastro-oesophageal reflux: 14% vs. 11% No aspiration pneumonia in either gp
	2. OI (n = 226)		NR				
Studies in which age category is unclear							
Bufano et al., 1990 [49]	1. NG (n = 9)	25% estimated req.	25% req	100% req. (2311 ± 607)	N	Early anthropometry: NR n = 3 (33%): Baseline ↓ K normalised after NG	- ↓ PO4 within 2 weeks of NG start: n = 3 (33%) - Other electrolytes within range during refeeding - No gastrointestinal symptoms reported by participants

^a^ unless otherwise specified; * significant difference; ↑ increase; ↓ decrease; AN: anorexia nervosa; AN-BP: anorexia nervosa binge-purge subtype; AN-R: anorexia nervosa restrictive subtype; BN: bulimia nervosa; BMI: body mass index; BP: binge-purge episodes; CBT: cognitive behavioural therapy; CHO: carbohydrate content; ETN: enteral tube nutrition; FFM: fat-free mass in kg; FM: fat mass in kg; Gp: group; K: potassium; LOS: length of stay; Mg: magnesium; N: no; NG: nasogastric nutrition group with at least some nutrition via NG; NR: not reported; n.s: no significant difference; OI: oral intake group; ONS: oral nutritional supplements; PO4: phosphate; req: requirements; RFS: refeeding syndrome; RH: refeeding hypophosphataemia; subgp: subgroup; Y: yes.

**Table 3:** Summary of overall nutrition and outcomes relate to anthropometry and physiology.

Author, Year	Overall Nutrition			Outcomes		
	Cohort (s)	*Target Nutrition:* (kcal/d or kcal/kg/d) Mean ± SD *Median (IQR, Range)*	*Duration of ETN (Days)* Mean ± SD (Range) *Median (IQR)*	*Length of Stay (LOS) (Days)* Mean ± s.d *Median (IQR)*	*Anthropometry* *Weight: kg* *Weight Gain per Week: kg/week* *BMI: kg/m^2^* *%EBW/IBW/BMI: %* Mean ± SD *Median (IQR, Range)*	*Physiological*
Studies in participants < 18 years of age						
Agostino et al., 2013 [6]	1. NG 2. OI	1.: 2276 ± 123 2.: 2373 ± 220	NR	1. vs. 2.: 33.8 ± 11 vs. 50.9 ± 24 *	1. vs. 2.: Weight gain over LOS: 4.44 ± 2 vs. 4.81 ± 2.3	-
Blikshavn et al., 2020 [42]	1. NG-R 2. no NG-R	1. and 2.: NR	NR Majority of NG-R episodes in first 8 weeks	Overall: 142 ± 95.9. 1.: LOS NR	Overall: Weight gain over LOS: 7.4 ± 4.5. BMI adm vs. d/c: 15.2 ± 1.9 vs. 18.3 ± 1.7. 1. vs. 2.: weight gain, BMI, BMI percentile change n.s.	-
Madden et al., 2015a [30]	1. NG	1.: 2400–3000	Aiming for max.: 15	28.62 ± 15.28	Overall weight gain over LOS: 5.12 ± 2.96 % EBW baseline vs. at 2.5 weeks: 78.37% ± 6.5 vs. 85.58% ± 6.46 * Gain of 7.21% ± 3.70 over 2.5 week	-No hypoglycaemia seen during treatment. -Refeeding electrolyte results as per initial nutrition outcomes.
Madden et al., 2015b [20]	1. Medical stabilisation w/NG 2. Weight restoration w/NG	1. and 2.: 2400–3000	Aiming for max.: 15	1. vs. 2.: 21.73 ± 5.92 vs. 36.89 ± 17.06 * 1.: d/c on med stab. 2.: d/c after weight restoration.	1. vs. 2. %EBW at hospital discharge: 84.4% vs. 92% *	-
Marchili et al., 2023 [43]	1. NG 2. OI	1. and 2.: NS	*21 (13)*	1. vs. 2.: 30 ± 11 vs. 16 ± 9 *	BMI adm vs. d/c: 1. 14.5 ± 1.9 vs. 15.5 ± 1.7 2. 16.1 ± 2.8 vs. 16.7 ± 2.8 (1.vs 2. BMI at adm *, BMI at d/c *)	-
Nehring et al., 2014 [50]	1. NG 2. OI	1. and 2.: NS	NR	1. vs. 2.: LOS longer in NG gp *.	-	-
Pruccoli et al., 2021 [51]	1. E AAP + E NG 2. E AAP + L NG 3. L AAP + E NG 4. L AAP + L NG 5. NG only	NS	7 (at least) Early NG start: ≤7 days of adm.	1. vs. 3. and 4.: LOS: 81.3 ± 31.4 vs. 182.6 ± 110.9 * and 155.4 ± 56.4 *, respectively.	Overall BMI: adm vs. d/c: 13.7 ± 1.7 vs. 15.6 ± 1.7 Early vs. late AAP: BMI change. n.s. Early vs. late NG: BMI change n.s.	-
Pruccoli et al., 2022 [52]	1. NG 2. OI	1. and 2.: NS	NR	NR	Overall: BMI adm vs. d/c: 14.3 ± 1.7 vs. 16.0 ± 1.6 6-month FU BMI (for those with available data, i.e., the ‘good outcome’ group): 17.3 ± 2.3	-
Pruccoli et al., 2024 [26]	1. NG 2. other	RFS gp: 1378 ± 289. No RFS gp: 1357 ± 231	NR	NR	Admission vs. d/c %mBMI: RFS gp: 71 ± 10 vs. *79 (11)* No RFS gp: 72 ± 10 vs. *80 (13)* Admission vs. d/c BMI: RFS gp: *14 (2)* vs. *15 (2)* No RFS gp: *14 (3)* vs. *16 (3)*	-RFS results as specified in the initial nutrition table: -NG use similar in the RFS and No RFS groups
Robb et al., 2002 [36]	1. NG 2. OI	1. and 2.: Target weight gain: 1–2 kg/week 1. max intake: 3255 ± 668 2. max intake: 2508 ± 478	NR	1. vs. 2.: 22.3 ± 13.5 vs. 22.1 ± 9.4.	1. vs. 2.: Weight gain over LOS: 5.4 ± 4.0 vs. 2.4 ± 1.8 * BMI change over LOS: 2.03 ± 1.4 vs. 0.9 ± 0.7 * d/c BMI: 17.5 ± 1.3 vs. 16.8 ± 1.6. n.s.	-
Silber et al., 2004 [24]	1. NG 2. OI	1. max energy intake: 4350 2. max energy intake: 3400	NR	1. vs. 2.: 36 ± 11 vs. 39.9 ± 22	1. vs. 2.: Weight gain over LOS: 10.9 vs. 3. BMI change 3.75 ± 2.3 vs. 1.2 ± 0.8. 1. BMI admission vs. d/c: 15.3 ± 1.7 vs. 19.1 ± NR 2. 17.4 ± 2.3 vs. 18.5 ± NR	-
Trovato et al., 2022 [45]	1. NG + ONS + OI 2. ONS + OI 3. OI 4. NG + OI	All groups: At least **40 kcal/kg/d** If weight loss ↑ to **50 kcal/kg/d**	NR	Overall: 27.5 (14–40) 1. vs. 2. LOS shorter * 1. vs. 4. LOS shorter *	NR d/c when weight restored.	-
Studies in participants ≥ 18 years of age						
Braude et al., 2020 [46]	1. NG 2. OI	1. and 2.: NR	*6 (2–12)*	Overall: 9.6 (5.8–19.7). BMI < 16 vs. >16: 11.7 (6.7–20.8) vs. 7.8 (5.3–15.9) *	Overall: Weight gain over LOS: 1.4 ± 2.9 1. vs. 2. anthropometry: NR	-
Hanachi et al., 2013 [33]	1. ↑ AST/ALT w/ETN gp 2. ↔ AST/ALT w/ETN gp	1. 1069 ± 350 via NG. Via OI: NS 2. 1763 ± 510 * via NG Via OI: NS	28 (study length)	NR	1. vs. 2.: lower weight gain in group 1 * BMI ↑: 1.5 ± 1.0 vs. 2.0 ± 0.8 *	1. n = 52/54 had normalised LFTs by week 4 of ETN. 2. no development of deranged LFTs during refeeding via ETN.
Martini et al., 2024 [44]	1. NG 2. OI	1. 1501 ± 369 2. 1421 ± 321	25 ± 19	1. vs. 2.: 39 ± 23 vs. 36 ± 20.	BMI adm vs. d/c: 1. 13.99 ± 1.76 vs. 14.80 ± 1.63 2. 14.01 ± 1.89 vs. 14.51 ± 1.69 BMI increase: 1. vs. 2.: 0.81 ± 0.74 vs. 0.55 ± 0.69 *	-
Rigaud et al., 2010 [40]	1. Low sodium gp w/NG 2. Std sodium gp w/NG	1.: 2647 ± 192 (862 ± 92 via NG). 2.: 2684 ± 186 (812 ± 109 via NG).	60 (at least)	1. and 2.: 60 (at least)	Month 1: True weight gain vs. expected weight gain: 1. 3.8 ± 0.3 vs. 4.0 ± 0.4 n.s 2. 6.7 ± 0.5 vs. 3.9 ± 0.3 * Overall, at BMI 15–16: n = 192 (88%)—weight ↔ for 7–10 days despite no change in energy input.	Overall: Urinary sodium output ↑ at 4–6 weeks of refeeding
Rigaud et al., 2007 [18]	1. NG 2. OI	1. and 2.: Prescription to achieve target weight gain: 1 kg/week	60 (at least)	1. and 2.: 67–70	1. vs. 2.: Weight gain in 8 wks: 9.6 vs. 5.9 * Weight gain/week over study period: 1.358 vs. 0.882 * FFM gain and FM gain higher in NG gp * BMI of 18.5 achieved at d/c: n = 16 (39%) vs. n = 3 (8%) *	-
Rigaud et al., 2011b [19]	1. NG + CBT gp (AN subgp) 2. OI + CBT gp (AN subgp)	1. and 2.: In AN subgp: target weight gain: 1.5–2 kg/month.	60 days (at least)	n/a (outpatient study)	In AN subgps: 1. vs. 2.: FFM at 8 weeks higher in NG gp *, remained higher at 6 months FU *. BMI adm vs. 8 wk in AN (both gps): 1. 15.8 ± 1.3 vs. 17.4 ± 1.1 * 2. 16.2 ± 1.0 vs. 16.6 ± 0.9	
Studies in participants across both age categories						
Born et al., 2015 [37]	1. PEG 2. No PEG	1.: Max. 3000 2.: NR	NR	Overall: 150.2 ± 80.8. range: 56–348. 1. vs. 2.: longer *	Overall: Weight gain over LOS: 12 ± 5.1. BMI adm vs. d/c: 12.3 ± 1.4 vs. 16.7 ± 1.7. 1. vs. 2.: weight gain over LOS: 12.2 ± 5.2 vs. 11.3 ± 4.7 n.s	-
Gentile, 2012 [38]	1. NG	1.: ~2800	90	NR	Weight gain/week: 1.1 ± NR * Adm vs. day 90: Weight: 27.9 ± 3.3 vs. 43 ± 5.7 * BMI: 11.2 ± 0.72 vs. 17.3 ± 1.6 *	n = 1 (10%)—hypoglycaemia on day 90—after NG cessation
Gentile et al., 2008 [47]	1. NG 2. OI	1.: Energy via NG: 1375 ± 211. Energy via OI NS. 2.: NS	133.8 ± 76	1. vs. 2.: 222 ± 136.9 vs. 164.3 ± 118.6	1. vs. 2.: Weight gain over LOS: 15.9 ± 3.4 vs. 14.0 ± 2.3 Adm and inpatient d/c BMI similar in both gps	-
						
Kells et al., 2022 [25]	1. NG 2. OI	1. and 2.: 1000–3000	NR	Overall: 7.4 ± 5.9	Overall weight gain over LOS: 1.8 ± 1.5 1. vs. 2.: weight gain similar n.s.	Refeeding electrolyte results as per initial nutrition outcomes.
Kezelman et al., 2018 [31]	1. NG	1.: 2400–3800	NR	24.81 ± 12.51	BMI admission vs. d/c: 16.32 ± 1.94 vs. 19.36 ± 1.15 *	-
Minano Garrido et al., 2021 [35]	1. ETN	1.: **50 kcal/kg/d**	35 (duration of study)	35 (at least)	Baseline vs. end of study: BMI: 11.4 ± 1.3 vs. 12.6 ± 1.02 * Total muscle strength, proximal muscle strength: ↑ with time * Axial muscle strength, distal muscle strength: n.s.	Baseline vs. end of study: Peak expiratory flow ↑ *, although still below normal range.
Murciano et al., 1994 [39]	1. NG	1.: Average ETN: Day 7-day 30: *1841 ± 519.* Day 30–45: *1567 ± 527* OI energy: NR	42 (at least)	56 (at least)	Day 0 vs. Day 30 and day 45 of refeeding: Weight: 37.1 ± 4.7 vs. 41.8 ± 4.8 * and 42.9 ± 4.6 *. Muscle mass (kg): 11.2 ± 4.1 vs. 15.1 ± 4.6 * and 16.6 ± 4.9 *. Fat free mass (kg): 33.3 ± 3.7 vs. 36.1 ± 3.1 * and 36.3 ± 2.9 * Fat mass (kg): 3.5 ± 1.5 vs. 5.7 ± 2.1 * and 6.5 ± 2.3 *	-Diaphragmatic function ↑ at Day 30 * and Day 45 * vs. Day 0 -FEV1 ↑ at Day 30 * vs. Day 0
Paccagnella et al., 2006 [32]	1. NG	1.: ~1450	20.7 ± 7.1	NR.	Weight: early refeeding: 33 ± NR, later refeeding: 40.4 ± NR, FU: 46.1 ± NR. BMI: early refeeding: 12.9 ± NR, late refeeding: 15.8 ± NR. FU: 17.3 ± NR	-
Parker et al., 2021 [10]	1. Low CHO w/NG 2. Standard CHO w/NG	1.: *3350 (3188, 3350)* 2.: *3325 (2844, 3350)*	1. and 2.: 7 (at least)	1. vs. 2.: 24.3 ± 11.3 vs. 24.4 ± 6.5	1. vs. 2.: weight gain by week 3: 6.5 ± 2.3 vs. 6.4 ± 2.0. 1. and 2: %mBMI ↑ over time *	1. vs. 2.: No difference in hypoglycaemia incidence. RFS parameters as per initial outcomes.
Rigaud et al., 2012 [34]	1. NG	1.: Week 3: **45 kcal/kg/d** (maintenance) Week 4: → target weight gain: 0.7–1 kg/week	21 (at least)	NR	Baseline vs. day 28: Weight: 25.9 ± 1.4 vs. 30.5 ± 1.2 BMI: 10.1± 0.28 vs. 12.1 ± 0.2 FFM: 24.1 ± 1.1 vs. 27.4 ± 1.0 FM: 1.6 ± 0.9 vs. 3.1 ± 0.9	-
Zuercher et al., 2003 [41]	1. NG 2. OI	1. and 2.: Target weight gain → >1 kg/week	36 ± 21	1. vs. 2.: 60.8 ± 17.3 vs. 48.3 ± 19.4 *	1. vs. 2.: Weight gain mean diff. over LOS: 8.1 vs. 5.7 weight gain/week: 0.91 vs. 0.82 NG > ½ LOS vs. NG < ½ LOS vs. OI: Weight gain/week: 1.0 vs. 0.77 * vs. 0.82 * MUAC higher * if NG > ½ LOS compared with NG < ½ LOS and OI gps.	1. vs. 2.: No difference in medical complications when controlled for severity of illness
Age category unclear						
Bufano et al., 1990 [49]	1. NG	1.: 100% requirements. 2311 ± 607	21 ± 14	NR	Weight gain/month: 8.22 ± 3.43 Tricipital and MUAC. ↑ *	-ALT, AST ↑ with NG: n = 9 (100%) → normalised within 1–2 weeks after stopping NG. -n = 2: ‘intense’ ↑ in appetite → normalised ~ 1 month post d/c

* significant difference; ↑: increase; ↔: normal range; AAP: atypical anti-psychotics; adm: admission; ALT: alanine transaminase; AN: anorexia nervosa; AST: aspartate transaminase; BMI: body mass index; BMR: basal metabolic rate; CBT: cognitive behavioural therapy; CHO: carbohydrate content; d/c: discharge; diff: difference; E AAP: early atypical antipsychotics start; %EBW: %expected body weight; E NG: early NG start; ETN: enteral tube nutrition; FEV1: forced expiratory volume 1; FFM: fat-free mass; FM: fat mass; FU: follow up; Gp: group; %IBW: % ideal body weight; K: potassium; L AAP: late atypical antipsychotics start; LFTs: liver function tests; L NG: late NG start; LOS: length of stay; %mBMI: % median BMI; Mg: magnesium; MUAC: mid-upper arm circumference; NG: nasogastric nutrition group; NG-R: NG under restraint; n.s: no significant difference; NR: not reported; OI: oral intake group. ONS: oral nutritional supplements; PEG: percutaneous endoscopic gastrostomy nutrition; PO4: phosphate; RH: refeeding hypophosphataemia; w/NG: with NG.

**Table 4:** Summary of relapses, readmissions, and longer-term outcomes.

Author, Year	Cohort (s)(n).	Readmissions Mean ± SD *Median (IQR)*	Longer-Term Outcomes Mean ± SD *Median (IQR)* Occurrence: n (%)
Studies with participants < 18 years of age			
Agostino et al., 2013 [6]	1. NG (n = 31) 2. OI (n = 134)	1. vs. 2.: readmission rates at 6 months.: n = 4 (12.9%) vs. n = 31 (23%)	-
Blikshavn et al., 2020 [42]	1. NG-R (n = 8) 2. no NG-R (n = 30)	1. vs. 2. at 5-year FU: Readmissions: n = 5 (63%) vs. n = 9 (30%) n.s. (medium effect size)	1. vs. 2. at 5 yr FU: ED diagnosis: n = 6 (75%) vs. n = 10 (33.3%) * BMI: *18.5 (15.6–20.8)* vs. *19.7 (19–20.7)* n.s. (medium effect size)
Madden et al., 2015a [30]	1. NG (n = 78)	-	At 12-month FU: % EBW: 95.33% ± 9.47 *
Madden et al., 2015b [20]	1. Medical stabilisation. gp w/NG (n = 41) 2. Weight restoration gp w/NG (n = 41)	1. vs. 2.: Hospital days over 12 months, post initial d/c: 22.78 ± 41.59 vs. 27.51 ± 51.70 Overall hospital days (including initial admission) lower in 1. * Readmission rates: 36.1% vs. 33.3% Reasons for admissions similar in both groups (malnutrition, self-harm)	1. vs. 2.: %EBW change admission to 12-month FU: 17.77 ± 11.36 vs. 15.75 ± 9.24. n.s. Full remission at 12-month FU: 30% vs. 32.5%. n.s.
Marchili et al., 2023 [43]	1. NG (n = 101) 2. OI (n = 214)	1. vs. 2.: Relapse: n = 14 (13.9%) vs. n = 33 (15.4%) Time to recurrence of symptoms median (IQR): 8.2 (9.7) vs. 3.0 (6.0) months *	
Nehring et al., 2014 [50]	1. NG (n = 71) 2. OI (n = 137):	1. vs. 2.: No. of hospital admissions higher in 1. *	1. vs. 2.: At FU (mean 6 years): BMI and height (marker of growth) →no difference
Pruccoli et al., 2022 [52]	1. NG (n = 33): 2. OI (n = 43):	-	Good outcome gp (completed initial admission, available for FU at 6 months and %mBMI > 70% at FU) vs. treatment resistant gp: NG use in admission: n = 23 (50%) vs. n = 10 (33.3%) * Treatment resistance associated with older participants, higher EDI 3 EDRC scores and less NG use *
Studies with participants ≥ 18 years of age			
Braude et al., 2020 [46]	1. NG (n = 27) 2. OI (n = 68)	Overall readmissions rate: 57.9% (timescale NR) Readmissions within 3 months: 30.3% Lower BMI: → longer initial admission * and lower readmissions in 3 months *	-
Rigaud et al., 2007 [18]	1. NG (n = 41) 2. OI (n = 40)	1. vs. 2.: Time before relapse in weeks: 34.3 ± 8.2 vs. 26.8 ± 7.5 * % relapsing patients at year 1: 44% vs. 52% Energy, lipids, CHO, protein intake ↓ with time over 1 year in both gps. *	-
Rigaud et al., 2011b [19]	1. NG + CBT (n = 52, AN subgp: n = 19) 2. OI + CBT (n = 51, AN subgp: n = 17)	-	1. vs. 2. BP episode ↓ of >75%: At 3 months FU: n = 46 (88%) vs. n = 23 (45%) * At 12 months FU: n = 43 (82%) vs. n = 23 (45%) * Similar results in AN and BN.
Studies with participants across the age categories			
Born et al., 2015 [37]	1. PEG (n = 57) 2. No PEG (n = 11)	Overall: Readmissions: n = 7 (10.3%) Duration of follow-up—NR	-
Gentile et al., 2008 [47]	1. NG (n = 32) 2. OI (n = 67)	-	In those completing the study (n = 75): Weight gain rate at outpatient d/c vs. inpatient d/c: 1.4 ± 1.1 kg/month vs. 3.1 ± 1.6 kg/month
Rigaud et al., 2011a [48]	1. NG (n = 262) 2. OI (n = 222)	Overall relapse rate: At year 1: 52.1% At year 2: +16.4% ETN > 2 months –↓ relapse at 2-year FU *.	Overall (13-year FU): n = 292 (60.3%) recovered. n = 125 (25.8%) ‘good outcome’. n = 31 (6.4%) ‘bad outcome’. n = 31 (6.4%) ‘severe outcome’. n = 6 died.
Rigaud et al., 2012 [34]	1. NG (n = 41)	n = 12 (29%): readmitted >4 times	6-year FU: n = 15 (36.6%) recovered; n = 11 (27%) ‘good outcome’; n = 6 (14.6%) ‘poorer outcome’; n = 6 (14.6%) ‘severe outcome’; n = 2 (4.9%) died

* significant difference; ↓ decrease; AN: anorexia nervosa; BMI: body mass index; BN: bulimia nervosa; BP: binge-purge; d/c: discharge; CBT: cognitive behavioural therapy; CHO: carbohydrate; % EBW: percentage expected body weight; ED: eating disorder; EDI: Eating Disorder Inventory; EDRC: Eating Disorder Risk Composite; ETN: enteral tube nutrition; FU: follow up; gp: group; %mBMI: percentage median BMI; NG: nasogastric tube feeding group; NG-R: NG feeding under restraint; NR: not reported; NS: not specified; n.s: not statistically different; OI: oral intake group; subgp: subgroup; w/NG: with NG nutrition.

**Table 5:** Transition from ETN to OI and outcomes.

Author, Year	Cohort (s)	Transition from ETN Towards Increasing Oral Nutrition				Outcomes During Transition Weight: kg BMI: kg/m^2^ Mean ± SD Occurrence: n (%)
		Basis for OI Introduction	When Transition Began	Strategies Used: Energy: kcal/d Median (Range)	Duration of Transition	
Studies in participants < 18 years of age						
Agostino et al., 2013 [6]	1. NG 2. OI	Med stab. + target energy reached, or NG completed for 7 days. NG only: max. 7 days	On day 7 at least	As OI ↑, NG titrated ↓. When NG ceased: NS	Over 3 days	1. vs. 2.: weight gain/week in days 1–14 incorporating transition: 1.06 ± 0.9 vs. 0.69 ± 0.6 1.: NG days 1–7 (mostly NG) vs. NG days 1–14 (incorporating transition): weight gain/week: 1.22 ± 1 vs. 1.06 ± 0.9
Kezelman et al., 2018 [31]	1. NG	When med stab	NS	As OI ↑, NG nocturnal and titrated ↓. OI staged plans from 1800 to 3800 kcal/d as required.	NS d/c when no NG required and BMI ≥ 18.5 kg/m^2^	-
Madden et al., 2015a [30]	1. NG	When med stab	Days 1–7 of admission	↑ OI during the day with nocturnal NG. Energy prescription: Days 1–7: NG 1000. OI 1500–1800 Days 8–14: NG 500 OI 2100–2400 Day 15 onwards: aiming for OI only 2400–3000	Aiming: by day 15	Weight gain: Week 0.0–0.5 (NG predominant): 1.78 ± 1.78 Week 0.5–1 (OI predominant): 1.01 ± 0.81 Week 1–1.5 (NG ↓, OI ↑): 0.51 ± 0.59. Week 1.5–2 (NG further ↓, OI ↑): 0.22 ± 0.48 Week 2–2.5 (NG stopped, OI only): 0.22 ± 0.62
Madden et al., 2015b [20]	1. MS w/NG 2. WR w/NG	When med stab at daytime.	NS	↑ OI started with daytime med stab till day 15 when aiming for full OI. 1.: full med stab maintained for 72 hrs—d/c 2.: after full med stab., continue target weight restoration at 1 kg/week until weight restored.	Aiming: by day 15	-
Robb et al., 2002 [36]	1. NG 2. OI	OI from the start. Basis of transition towards ↑ OI and ↓ NG NS.	NS	As OI ↑ NG titrated ↓. by 2 hrs/night (300 kcal). NG cessation: when 95% IBW reached, OI at maintenance req. →d/c after 3–4 days.	NS	-
Silber et al., 2004 [24]	1. NG 2. OI	OI from the start. Basis of transition towards ↑ OI and ↓ NG NS	NS	As OI ↑ NG titrated ↓. NG cessation: when OI → at maintenance req. d/c 1–2 days after.	NS	-
Trovato et al., 2022 [45]	1. NG + ONS + food 2. ONS + food 3. food only 4. NG + food	OI 1st line. If OI < 70% intake ONS introduced. If OI + ONS < 30% intake NG introduced. NG ↓ based on weight ↑.	NS	As weight ↑ NG ↓.	NG ↓ over 6–7 days. 4/5 of NG for 2 days 3/5 of NG for 2 days 2/5 of NG for 2 days.	-
Studies in participants ≥ 18 years of age						
Rigaud et al., 2010 [40]	1.Low sodium w/NG 2. Standard sodium w/NG	OI from the start. Basis of transition towards ↑ OI and ↓ NG: NS	NS	1.: low sodium OI changed to standard sodium OI when urinary sodium clearance improved ~4–6 weeks. NG ↓: NS	NS NG:OI: Month 1: 1:1.7 Month 2: 1:2.2	-
Rigaud et al., 2007 [18]	1. NG (intervention arm) 2. OI (control arm)	OI from the start. Encouraged to ↑ OI.	NS	As OI ↑, NG titrated ↓. Target weight gain: 1 kg/week.	NS NG: OI Month 1: 1:2.7 Month 2: 1:3.3	1. vs. 2.: OI ↑ similar. n.s. 1.: OI ↓ and weight ↓ when NG stopped: n = 4 (9.8%). OI ↑ 1 week after NG stopped: n = 25 (61%)
Rigaud et al., 2011b [19]	1. NG + CBT (AN subgp: n = 19) 2. OI + CBT (AN subgp: n = 17)	NS	OI introduced in week 4.	OI introduction in steps every 2–3 days: Step 1 breakfast, step 2 lunch, step 3 dinner. As OI ↑, NG titrated ↓	5 weeks (from week 4 to 8) Duration of NG: 8 weeks.	-
Studies in participants across both age categories						
						
Born et al., 2015 [37]	1. PEG 2. No PEG	OI from the start. Basis of further transition: NS	NS.	OI encouraged. ETN continued until BMI > 17 kg/m^2^ + able to maintain weight with OI for 2 weeks	NS	-
Gentile 2012 [38]	1. NG	NG duration ↓ with weight ↑. Basis of transition towards ↑ OI and ↓ NG: NS.	~Day 30 of refeeding	↑ OI with ↓NG: Energy prescribed: Day 30: NG 1100 OI 1412 Day 60: NG 1000 OI 1819 Day 90: NG 800 OI 2135	~2 months. NG stopped after Day 90	BMI Day 30 vs. Day 90: 14.1 ± 0.9 vs. 17.3 ± 1.6 BMI ↑ mean diff in BMI pts: Day 0 to Day 30 (NG > OI): 2.9 BMI pts in 1 month. Day 30 to Day 90 (OI > NG): 3.2 BMI pts in 2 months. Weight gain: Day 0 to Day 30 (NG > OI): 7 kg in 1 month. Day 30 to Day 90 (OI > NG): 8.1 kg in 2 months. n = 1: severe hypoglycaemia after stopping NG
Gentile et al., 2008 [47]	1. NG 2. OI	OI encouraged from the start. ↑ OI → when past life-threatening stage + agreeing to ↑ OI	NS	OI encouraged.	NS.	-
Paccagnella et al., 2006 [32]	1. NG	OI introduced on days 3–4.	Days 3–4	↑ OI encouraged. When OI >50% energy requirements, NG ↓. At d/c OI only. Average intake at d/c: ~1450 kcal/d	NS. NG duration: 20.7 ± 7.1 days	Abnormal baseline ALT improved in n = 5 (out of n = 7), others (n = 2) improved within 3 weeks of stopping NG.
Parker et al., 2021 [10]	1. Low CHO feed 2. Standard CHO feed	When med stab.	Med stab in both gps: at day 2 On average OI introduced on Day 3.	Staged OI plans between 1800 and 3800 kcal/day. Replacement ONS as needed. As OI ↑, NG, nocturnal and titrated ↓. At week 1: energy via OI vs. total energy: 1.: 2300 (1800, 2300) vs. 3350 (3188, 3350). 2.: 2300 (1800, 2300) vs. 3325 (2844, 3350). NG cessation: NS.	NS NG continued until week 3. NG:OI: Week 2: 1:4 Week 3: 1:6.5	1. vs. 2.: Weight gain by week 1 (NG predominant in 0–0.5 week): 2.7± 1.9 vs. 2.7 ± 1.6 n.s. Weight gain by week 2 (OI predominant 0.5–2.0 week): 4.9 ± 1.9 vs. 4.6 ± 1.5. n.s. Weight gain by week 3 (higher proportion OI): 6.5 ± 2.3 vs. 6.4 ± 2.0 Weight gain/week ↓ with time.
Rigaud et al., 2012 [34]	1. NG	Protocol based	Days 11–21: OI introduced. Timing of NG ↓: NS	OI introduced as small meals (300–400 kcal). From week 4—target weight gain: 0.7–1 kg/week. As OI ↑, NG titrated ↓. NG cessation: NS	NS	BMI ↑ mean diff.: Days 1–2 (NG) vs. Days 13–15 (OI introduced): 1.2 BMI pts Days 13–15 vs. Days 28–32 (NG ↓): 0.8 BMI pts. Weight gain mean diff.: Days 1–2 (NG) to days 13–15 (OI introduced): 2.8 kg Weight gain days 13–15 to days 28–32: 1.8 kg
Zuercher et al., 2003 [41]	1.NG 2. OI	OI from the start. Basis of transition towards ↑ OI and ↓ NG: NS	NS.	NG cessation: with enough OI practice prior to d/c.	NS Duration of NG: 36 ± 21 days	-
Age category unclear						
Bufano et al., 1990 [49]	1. NG	When participant asked for OI to start.	NS	NG stopped when OI > (BMR +30%) for ≥3 consecutive days	NS	n = 2: spontaneous ↑ in OI to >3000 kcal/d, ↑ number of meals & ↑ CHO: P intake. Lasted for ~1 month post d/c

↑: increase; ↓: decrease; ALT: alanine transaminase; BMI: body mass index; BMR: basal metabolic rate; CBT: cognitive behavioural therapy; CHO: carbohydrate content; d/c: discharge; diff: difference; ETN: enteral tube nutrition; Gp: group; hrs: hours; %IBW: % ideal body weight; med. stab.: medical stabilisation. MS: medical stabilisation group; NG: nasogastric tube nutrition; NS: not specified; n.s: no significant difference; OI: oral intake; ONS: oral nutritional supplements; P: protein; PEG: percutaneous endoscopic gastrostomy nutrition; pts: points; req: requirement; subgp: subgroup; w/NG: with nasogastric tube nutrition; WR: weight restoration group.

### 3.2. Outcomes Related to ETN During Initial Nutrition

#### 3.2.1. Anthropometric Outcomes

Reported weight gain in the initial refeeding phase of ETN (Table 2) was between 0.73 and 2.79 kg/week [6,10,25,30,34,38,39,40]. Weight gain with a conservative energy prescription in a study (1.2 kg in week one) [34] was comparable with a standard prescription in another study (1.22 kg in week one) [6]. Other studies with standard energy prescriptions reported weight gains of 2.0 kg [38] and 2.1 kg [39] in week one. Studies prescribing higher energy intakes reported gains of 2.7 kg [10] and 2.79 kg [30] in the first week of refeeding. Studies comparing ETN and OI cohorts found contrasting results with one reporting higher weight gain with the former group [6], while the other reporting similar weight gain in both groups [25], despite a significantly higher energy prescription in the ETN cohorts of both studies. Studies with higher energy prescriptions were in adolescent or young adult populations.

Overall, ETN and OI appeared to be comparable in achieving weight gain in this phase of refeeding. Furthermore, results suggested factors in addition to energy prescription affecting weight increase during this phase. A study comparing a standard sodium cohort with a low sodium intake group found a higher weight gain associated with the former, indicating that fluid balance related to sodium retention may be one such factor [40].

#### 3.2.2. Physiological and Psychological Outcomes

Results indicated improvement in function in this early phase of refeeding in studies across age categories. Diaphragmatic function improved within week one in one study [39], and neurological function by week three in another [34]. However, early refeeding was not associated with structural changes, such as muscle mass increases [39]. Hunger ratings improved in the first two weeks in one study [18]. In two RCTs in adults, binge-purge episodes improved in the ETN cohorts compared with the OI groups [18,19]. The ETN group reported increased anxiety in the first week of refeeding compared with the OI group in one study [18]. However, the anorexia nervosa binge-purge subgroup (AN-BP) in this cohort reflected on ETN being ‘useful’ which may have been related to a reduction in binge-purge episodes [18].

#### 3.2.3. RFS Parameters

##### Studies with Conservative Energy Prescriptions

The results regarding RFS parameters were heterogeneous (Table 2). Signs of mild RFS [32], refeeding oedema, and phosphate level reduction despite phosphate prophylaxis [34], were reported in two studies in participants across age categories. In contrast, an RCT in adults with an ETN and an OI arm supplemented with prophylactic phosphate reported no RFS, RH, or reduction in potassium levels during refeeding in either group [18]. A recent study in adolescents comparing groups presenting with or without RFS found that both groups had similar BMIs (mean BMIs: 14 kg/m^2^), a similar average energy intake, and similar ETN usage [26]. Overall, conservative energy prescription did not appear to mitigate RFS risk fully. ETN and OI routes appeared to have similar risks related to RFS. There appeared no differences in results based on age categories.

##### Studies with Standard Energy Prescriptions

Results from studies with the standard refeeding energy prescriptions were also heterogeneous. One study reported a lower incidence of RH in its ETN arm compared with its OI cohort [6]. However, most participants in the ETN arm had been given prophylactic phosphate which may have influenced the results. Additionally, a single ETN cohort, also given prophylactic phosphate, reported no reduction in phosphate levels during refeeding [38]. Both these studies used ETN as part of their refeeding protocol. Contrastingly, one study reported a three times higher risk of phosphate reduction in their ETN cohort compared with their OI group despite prophylactic phosphate being given [25]. In this study, ETN was administered as a replacement bolus when OI was refused (food and ONS). Baseline anthropometry was not significantly different between the ETN and OI groups; however, the ETN cohort had a higher energy provision overall [25]. Furthermore, another study, without prophylactic phosphate administration, reported that 33% of its participants required phosphate supplementation two weeks after starting refeeding [49]. These studies were conducted in adolescents [6], across the age categories [25,38] or where the age category was unclear [49] and no differentiation could be made in results based on age.

##### Studies with Higher Energy Prescriptions

A NG cohort (mean % Expected Body Weight (% EBW) 78%) starting refeeding at 2400 kcal/day, and an increasing energy prescription as required, with prophylactic phosphate given, reported no incidence of phosphate reduction in its participants [30].

An RCT with a rapid increase in energy prescription, comparing arms with a low-carbohydrate feed and a standard carbohydrate feed, reported a significantly lower incidence of phosphate reduction in the low-carbohydrate feed arm. Requirements for potassium and magnesium supplementation due to lowering levels were not significantly different between the groups [10]. This study only provided prophylactic phosphate if levels were ≤1 mmol/L prior to refeeding.

These studies were conducted in adolescents [30], or in adolescents and young adults [10], with no studies in an exclusively adult population.

##### RFS—Other Parameters

With regard to other RFS parameters, a study in adults in which ETN was provided when participants were unable to comply with OI or were unwell and at a higher risk reported a higher incidence of hypokalaemia but not RH or hypomagnesaemia [46]. ETN use and electrolyte derangements were not associated with BMI (mean 17.1 kg/m^2^) in this study [46]. Contrastingly, another study in participants across age categories with a mean BMI of 16.3 kg/m^2^, in which ETN was provided with OI refusal, found no difference in potassium or magnesium levels between ETN and OI cohorts [25].

##### RFS Parameters Summary

In summary, RFS-related derangements occurred in various contexts, including in studies with conservative or standard refeeding rates designed to mitigate RFS risk. While a lower BMI is said to increase RFS risk, studies with participants with a relatively higher BMI [25,46] given ETN were also associated with a reduction in phosphate and potassium levels, suggesting the need for a broader exploration of factors increasing RFS risk. Both these studies had ETN administered if OI was refused, or participants were more severely ill. Phosphate prophylaxis, in general, seemed to reduce the risk of RH. However, some studies highlighted that RH may be seen despite prophylaxis and that careful monitoring for the occurrence of RFS and supplementation of electrolytes when needed is of importance. Overall, ETN, as a nutritional route, did not appear to influence RFS risk. The use of low carbohydrate refeeding may support RFS risk reduction. However, more research is needed in this regard. No particular patterns were seen in age categories with RFS risk regarding energy prescription or route of nutrition. However, studies with higher ETN energy prescriptions had been conducted in adolescents or young adults only.

### 3.3. Overall Outcomes Related to ETN

#### 3.3.1. Anthropometric Outcomes

Weight gain outcomes were presented variably, although all ETN arms across studies showed weight gain from admission to discharge (Table 3). Some studies reported weight changes over the length of the study, ranging from a mean difference of 4.44 kg to 15.9 kg over a varied treatment period [6,10,24,30,36,47], approximating to a range of 0.5–2.2 kg/week. Other studies reported weight gain per week of treatment or study, ranging from 0.91 kg to 1.35 kg/week [18,38,41]. Two studies comparing ETN arms within each study found similar weight gain when both arms had feeds with equivalent energy prescriptions [10] and greater weight increases when one arm had a higher energy prescription than the other [33], as expected.

Cohort studies comparing ETN and OI arms showed either a similar weight gain [25,46,47] or a higher weight gain in the ETN arm compared with the OI arm [18,36,41]. Furthermore, in a cohort study, those given ETN for longer than half their stay had a higher weight gain per week compared with those with ETN for less than half their stay or those in the OI arm [41]. Additionally, a recent cohort study found a significantly higher BMI increase in its ETN group compared with the OI group [44]. This study matched participants who had received ETN with those who had continued with just OI through propensity scoring for confounders such as BMI, duration of illness, other psychiatric co-morbidities, Eating Disorders Examination Questionnaire (EDE-Q) scores, and number of hospitalisations, thus attempting to match the two groups as closely as possible.

Results were heterogeneous when comparing weight gain in ETN and OI arms in regard to energy prescriptions. Higher energy prescriptions in the ETN arms compared with OI arms either resulted in a higher weight gain in the ETN arm as expected [18,24,36] or similar weight gain in the ETN arm despite the higher energy prescription [25]. Contrastingly, one study reported a higher increase in BMI in the ETN arm despite similar energy prescriptions in both the ETN and OI arms [44].

In summary, included studies have shown consistent weight restoration in ETN arms, this being either higher or comparable with the OI arms. Studies have not shown a pattern related to the age of included participants. The heterogeneity of results suggests that factors, other than the mode of nutrition and energy prescription, such as acceptance of nutrition, presence of compensatory behaviours, and types of support provided, may also influence weight gain and need exploration within these contexts.

Studies reporting on fat-free mass changes described improvement over time in the ETN arms [18,19,39]. Mid-upper arm circumference (MUAC) and tricipital skinfold thickness increased significantly in a single cohort study [49]. MUAC was significantly increased in a group given ETN for more than half of their inpatient stay compared with those given ETN for less than half of their stay and those in the OI arm [41]. A study with a single ETN cohort reported increased muscle mass, fat-free mass, and fat mass at days 30 and 45 of admission compared with these measures at baseline [39]. A study comparing an ETN arm with an OI arm reported a significantly higher gain in fat-free mass and fat mass through two months of treatment in the ETN arm [18]. Structural changes were seen at the end of the study duration in these studies, lasting between 21 days and 60 days. Both fat mass and fat-free mass improvement correlated with weight restoration suggesting structural changes were driving these findings. Studies were predominantly in adults or across age populations.

#### 3.3.2. Physiological Outcomes

Physiological outcomes are presented in Table 3. Studies were predominantly in adults or across age populations. Liver function outcomes showed contradictory results. Two studies with abnormal baseline results, given ETN with a conservative prescription, had an improvement in liver function [33,35]. Two studies with normal baseline liver function showed contrasting results. One study reported normal results after ETN with a standard energy prescription [38], while another, also with a standard energy prescription, showed a worsening of liver function in participants, which improved on cessation of ETN [49].

Lung function was investigated in two studies. Peak expiratory flow improved at the end of one single cohort study [35], as did diaphragmatic function and forced expiratory volume 1 in another [39]. The former study also assessed muscle strength, which improved in patients given ETN, even with only partial weight recovery [35].

#### 3.3.3. Outcomes Related to Eating Disorder Symptoms and Other Psychological Outcomes

##### Eating Disorder Psychopathology

Psychological outcomes are presented in Supplementary Table S2. In terms of eating disorder symptoms, two RCTs in adults reported a significant reduction in binge-purge episodes in the ETN arms compared with the OI arms [18,19]. One study reported a continued significant reduction in these symptoms in its ETN arm in comparison with the OI arm at three- and 12-month follow-ups [19].

Eating disorder psychopathological overall scores showed improvement in both ETN and OI cohorts in studies across the age categories [18,19,20,41,44]. However, in a study using a rapid refeeding protocol in adolescents with AN, while global EDE-Q scores and Eating Concern and Restraint subscales improved with treatment, the Weight and Shape Concern subscales did not show a significant difference [31]. EDE-Q improvement was significantly related to treatment satisfaction in a study of adult participants [44]. Depression, anxiety, and self-esteem symptoms did not moderate the occurrence of full remission in adolescents in one study [20].

In summary, symptoms related to the eating disorder, for example, eating concern, restraint, and binge-purge episodes, appeared to improve with ETN. However, those specifically related to body image disturbance, such as weight and shape concerns, did not seem to change with nutritional treatment and weight restoration. Treatment satisfaction was associated with EDE-Q reduction, suggesting that participants associated the usefulness of AN treatment with psychological improvement.

##### General Psychopathology

Regarding broader psychopathology, a rapid refeeding protocol with ETN in an adolescent cohort was associated with improved measures of anxiety and depression. However, these measures were not significantly associated with BMI change over time [31]. One study in adults reported greater increases in Quality of Life scores in patients given ETN compared to OI over the course of treatment [19].

#### 3.3.4. Length of Stay Outcomes

##### Length of Stay When Comparing ETN and OI Cohorts

Results on length of stay were heterogeneous (Table 3). Some studies found that ETN was associated with a significantly longer admission compared with the OI group [41,43,50]. Other studies reported similar lengths of stays in the ETN and OI arms [24,36]. However, one study reported a significantly longer stay in the OI arm compared to the ETN arm [6]. This study provided a significantly higher energy prescription from the start for the ETN arm in comparison with the OI arm, which may have influenced the results. All studies were in adolescents [6,24,36,43,50] except for one study in participants across the ages [41].

##### Other Factors Influencing Length of Stay in ETN—Timing of Initiation and Adjunctive Treatment

A study comparing the use and timing of atypical antipsychotics (AAP) and of NG nutrition initiation, found that those who were given AAP and NG early had a significantly shorter stay than those with a late AAP and a late NG start [51]. Furthermore, a study reported a longer admission if NG, when required, was started more than 10 days after admission [43]. Regarding adjunctive nutrition, a study reported a shorter stay in a cohort given ONS alongside NG nutrition and food when compared with the cohort given NG nutrition and food only [45]. All these studies were in adolescent populations.

In summary, factors such as ETN use, the timing of ETN initiation, and whether ETN is used alongside ONS or combined with treatments such as AAPs, may influence the duration of admission. Most of this evidence was in adolescent populations.

#### 3.3.5. Readmissions, Relapses, and Remission

Variable results were reported regarding readmissions and relapses (Table 4). Two studies reported a longer time period before the recurrence of symptoms in the ETN cohort compared with the OI group [18,43]. Furthermore, another study reported lower relapse rates at the two-year follow-up in the cohort given ETN for two months compared with the OI group [48]. Readmission rates were reported as similar in the ETN and OI cohorts in one study [6]. Contrastingly, another retrospective study reported a higher number of admissions in the cohort that needed ETN in at least one admission [50]. Studies were heterogeneous in regard to age groups.

An RCT with both arms receiving ETN initially, testing the hypothesis that inpatient treatment until weight restoration would reduce readmission days, found that both cohorts—one with participants discharged after medical stabilisation and the other when participants were weight restored—had a similar number of readmission days, disproving their hypothesis [20]. Both cohorts had similar eating disorder remission rates at the one-year follow-up. Furthermore, a study with a 5.5-year follow-up found similar rates of remission in the ETN and OI cohorts [50]. Both these studies were conducted in adolescent populations.

In summary, when ETN was part of the protocol, either initially [6] or when specific OI parameters were not met [43] or were provided for at least two months [18,48], studies had better readmission and relapse outcomes associated with the ETN arms compared with OI cohorts. However, when ETN was required because of food refusal, the number of hospitalisations were higher [50], which may be indicative of the severity of the illness. The RCT by Madden et al. (2015b) [20] in medically unstable adolescents with AN suggested that longer admissions for weight restoration may not result in higher remission rates at the one-year follow-up.

### 3.4. Strategies Used for Transition from ETN to OI and Related Outcomes

Methods of transition from ETN towards more OI and outcomes are presented in Table 5. The protocols for the transition from ETN to OI were heterogeneous. OI in the ETN cohorts varied from being present from the start to being introduced with parameters of medical stabilisation. The duration of the transition phase varied from three days to three months. Indications for stopping ETN were based on acceptance of OI [6,24,41,49], anthropometric criteria [36], combinations of criteria [37], and cessation of ETN as part of the protocol for nutrition [10,20,30,38].

Anthropometric outcomes related to the transition phase were not compared with other phases, such as the ETN-only, predominantly ETN, or OI-only phases. However, trends in studies in adolescent [6,30] and mixed-age populations [38] indicated a deceleration of weight restoration during this phase compared with the ETN-only and the predominantly ETN phases. A study in adults described some participants (n = 5, 12%) in its ETN arm having a decrease in OI and showing weight loss following ETN cessation [18]. Difficulties in weight restoration may reflect barriers encountered in the acceptance of OI during the transition process. Factors influencing these outcomes within transition protocols need to be explored.

One cohort study in adolescents reported that the use of ONS alongside ETN and food intake resulted in a shorter length of stay compared with ETN and food intake alone [45]. This suggests that the use of ONS may support the transition process and hence the reduction in length of admission.

### 3.5. Qualitative Data and Their Synthesis

Six studies, two in adolescents or young adults [27,29] and four in adults [21,22,23,28], investigated qualitative data in ETN (Supplementary Table S3). The following areas were explored.

#### 3.5.1. Initiation of ETN

A theme emerging in participants starting ETN was the physical, undeniable presence of the tube with associated descriptors, such as ‘unpleasant’, ‘scary’, ‘feeling petrified’, or ‘traumatised’ [21,22,29], with these feelings intensified if participants were anxious or staff inexperienced [29]. Alienation from the decision-making process of ETN initiation [21] and the tube being associated with unfairness, punishment and a fight for control were described [22,28,29]. Conversely, clear communication about the rationale and the process of ETN and weight and discharge targets was described as helpful [27].

Benefits described were the tube as a means of recognising illness severity [27,29], a way of communicating their thoughts and feelings to their family [29], the relief of not having the responsibility for nutrition [28,29] and not having to taste or smell food [28], and, for some, a motivator to start OI [29].

#### 3.5.2. The Presence of Feed with or Without OI

In a study (participants aged 16–19 years) with a faster refeeding protocol with the expectation that daytime OI would be combined with nocturnal ETN, the participants reported that while the mechanics of eating became more manageable, the sensation of fullness, especially towards the end of the day, continued to be pronounced [27]. Conversely, in a study in adults, with NG nutrition only, participants felt that OI alongside ETN would have been beneficial in continuing the ‘habit of eating’ [21].

#### 3.5.3. Resisting ETN

In studies exploring the lived experience of NG under restraint, participants described alignment with the eating disorder, with physical restraint being a new standard, feelings of isolation from peers, feeling ‘stuck’ [22], experiencing internal coercion from the eating disorder and external coercion through involuntary treatment, and feeling trapped, hunted, and dehumanised [28]. Self-harm or purging after a feed could result in further restraint [28]. Resistance at the start subsiding through the admission with acceptance or learned helplessness was described by some [28]. Covert resistance was also expressed, such as tampering with feeds, negotiating ETN reduction with staff, and recruiting carers for ETN cessation [29].

#### 3.5.4. Transition Towards More OI

In one study, in which the protocol began with NG nutrition only, participants expressed worries about losing the habit of regular eating, with related difficulties transitioning back to OI [21]. Moreover, they compared themselves to those who were given food and expressed distress with exposure to food smells and not being allowed to eat [21]. Participants in a study with OI concomitant with ETN expressed the helpfulness of supported meals, with staff interacting with them rather than being ‘stared at’, during the transition to OI [27]. In this protocol, some described developing food aversions when expected to eat while needing NG nutrition. However, for those with a shorter duration of illness, the same protocol improved their enjoyment of food [27]. In one study of lived experience of NG nutrition under restraint, participants expressed feeling stuck and unable to move back to oral nutrition [22]. In some participants in another study, the tube was recognised as a part of their ‘self’ or as part of their eating disorder identity [29].

#### 3.5.5. A Life-Saving Measure

Participants acknowledged the need for ETN and its life-saving nature [21,22,28,29]. Upon reflection, knowing that NG under restraint would be administered if one did not comply with refeeding treatment, was seen as supportive of the battle against the eating disorder [28]. In retrospect, they were grateful for the life they were able to have afterwards [22,28]. Clinicians described meeting grateful patients who had been physically restrained for ETN in the past [22]. Looking back, participants described how they had lacked care towards themselves at the time, identifying that this had now changed, and they were not ‘self-destructive’ anymore [28].

#### 3.5.6. Trauma

Tube insertion and feeding were articulated as traumatic in the studies exploring NG under restraint [21,22,28]. Words used to describe this were ‘petrified’, ‘intrusive’, ‘haunted (by it)’, ‘smell…taste it’, and ‘an assault’ [22,28]. Hearing someone else being restrained for NG nutrition, carried them back to the time they had similar experiences [22]. Participants recalled being sensitive to sounds and receding into feeling like an animal hunted, leaving a lasting imprint on them [28]. Participants described re-experiencing the physical sensation of the tube insertion when they watched a NG insertion as part of their job or feeling retraumatised when needing to be tested for Coronavirus disease via nasal swabs. Some were diagnosed with post-traumatic stress disorder related to their experience [22]. Involuntary treatment was seen as a motivator, in retrospect, to never be in that situation again [28]. Parents and carers also described the trauma of hearing screams on the ward and imagining that it is their child being restrained for feeding. They, however, also expressed empathy for staff having to restrain patients as a life-saving measure [22].

#### 3.5.7. Ways of Improving the Experience of ETN

An explanation of the rationale, clear communication, and goal-setting for ETN cessation and for discharge were described as important parts of a collaborative treatment plan [21,23,27]. Individualised treatment plans, even within protocol-based treatment, were emphasised [23,27]. The importance of a safe space for the service users and carers to communicate their views and feelings was thought of as critical [23]. Establishing trust between the treatment team, service users, and carers was seen as a central part of this process [23].

## 4. Discussion

ETN is used in AN and AAN as a supportive measure needed in the context of food refusal and markers of medical compromise, such as those outlined in the Medical Emergencies in Eating Disorders guidance [5]. This systematic scoping review explored the use of ETN and associated outcomes in AN and AAN with a particular focus on transitions between ETN and OI.

The following questions were explored:

### 4.1. Is ETN a Viable and Safe Method of Providing Nutrition in AN and AAN?

This review found consistent improvement in anthropometry associated with ETN with conservative, standard, and higher energy prescriptions in this population. Both conservative and standard energy prescriptions produced similar weight gain initially, possibly influenced by fluid retention and sodium homeostasis. Studies with higher initial energy prescriptions followed by rapid increases in provided energy, all in adolescents or young people, showed higher weight gain per week and overall weight gain [10,20,30], part of which may have been related to fluid shifts, especially in early refeeding. Early and overall weight gain results seen here are in keeping with previous reviews, which found significant weight gain in the initial period with ETN in AN and an overall weight gain of 1 kg/week with NG nutrition [13,53]. When comparing ETN with OI, this review found either similar or higher weight gain in the ETN cohorts and is aligned with the findings of a previous review [12].

Regarding RFS parameters, most studies given prophylactic phosphate showed protection against hypophosphataemia. Overall, there was no evidence that ETN, when compared with OI, was associated with an increased RFS risk. Risks appeared similar in the different age categories. This largely aligns with evidence from previous reviews [12,53].

In summary, ETN, when required, is a viable alternative for supporting physical health improvement and does not appear to increase the risk of refeeding syndrome.

### 4.2. Does ETN Affect the Length of Admissions in AN and AAN?

The length of stay in ETN cohorts showed heterogeneous results in this review, with some studies showing a tendency towards longer admissions in ETN cohorts compared to OI cohorts. A previous review similarly reported that NG feeding was associated with a longer stay if ETN was in response to medical severity [54]. However, when ETN was part of a routine protocol, the length of stay was shorter than in OI-only cohorts [54]. The duration of ETN, and thus the length of stay, may be dependent on the severity of the illness and complexities in management. Additionally, if NG under restraint is required for a service user, this will likely mean a longer admission. Furthermore, decisions regarding discharge may be based on multiple factors, including discharge criteria of an eating disorder service, service user factors such as age, duration of illness, engagement with treatment, and preference to continue admission, as well as logistical factors such as the need for hospital beds, social care needs of the service user, and available follow-up support. These factors also need to be considered while analysing length-of-stay data. Regarding ETN, Pruccoli et al. (2021) [51] and Marchili et al. (2023) [43] highlighted the importance of clear decision-making in ETN initiation and the usefulness of adjunctive treatment in influencing admission length. Thus, a protocol or pathway for when ETN is indicated, including the process of initiation and cessation with some flexibility to personalise the pathway, with the involvement of the multidisciplinary team, service users, and carers [55], is indicated in supporting shorter inpatient stays.

### 4.3. What Are the Psychological Outcomes and Qualitative Data Related to ETN?

Overall, outcomes of the eating disorder and quality of life scores improved with treatment regardless of the route of nutrition [18,19,20,31,41,44]. Service users associated better quality of life and the perception of the usefulness of treatment with the reduction in eating disorder symptomatology [19,44]. Although eating concerns were reduced, weight concerns did not improve with nutritional treatment [31], suggesting the need for focused psychological support, such as body image work, in this context.

Qualitative data highlighted meanings attached by some service users to ETN, including being further aligned to AN with a firmer ‘AN identity’. Work to externalise the AN identity may be needed to mitigate these effects [56].

With regard to NG under restraint, a few service users may experience multiple episodes over long periods of time [42], indicating the need to explore risk factors associated with this intervention. The severity of illness and the need for NG under restraint may influence outcomes, such as the persistence of an eating disorder diagnosis during follow-up [42]. NG under restraint has been shown to be associated with long-term trauma. More research is needed for less restrictive supportive measures of nutrition, even when the eating disorder is severe [55]. Furthermore, research is also needed to proactively support service users in mitigating the effects of trauma.

### 4.4. How Is the Transition from ETN to OI Managed?

This review did not find any studies exploring the transition from ETN in AN and AAN. However, weight restoration slowed down during the transition period, which may indicate difficulties faced during this phase of refeeding. One study found that the cohort using ONS alongside NG nutrition and OI was associated with a reduced length of stay compared with NG and OI [45]. This may indicate the usefulness of ONS in the transition process.

A study of participants admitted to a medical ward for the treatment of restrictive eating disorders reported on the importance participants placed on having OI alongside NG nutrition [57], suggesting that this would be considered supportive of transition by service users. Another study found that the incidence of NG nutrition reduced from 67% to 11% when a protocolised meal support system was introduced in their unit [58]. It is possible that methods, such as changing from continuous to bolus ETN and reducing ETN for a few days, may also be helpful strategies, as described in a review exploring weaning from tube dependency in a paediatric population [59].

Qualitative data in this review indicate that the feeding tube could become a part of the ‘self’ and a signifier of illness [22,29]. This may inadvertently perpetuate the sick role and isolation of sufferers from their community. A planned exit strategy from ETN to OI may be indicated, with support provided to sufferers in finding their own identity outside of the eating disorder [56].

### 4.5. Strengths and Limitations of the Review

This scoping review explored outcomes in AN and AAN associated with ETN with a focus on transitions to and from ETN to OI. The study followed PRISMA guidance for scoping reviews and search strategies. Included studies were identified independently by two researchers, and any disagreements were resolved through discussion with co-authors and supervisors. The results were categorised according to study objectives.

However, the heterogeneity between studies in terms of a limited number of baseline characteristics, the content of nutritional protocols, and duration of ETN within them, and the outcomes measured made comparisons between studies difficult. This review only included studies published in English. Most studies were observational, and therefore, had associated biases. ETN may be used only when needed for medical stabilisation and food refusal, making outcome comparisons with cohorts that accept and continue with OI difficult. Studies researching ETN under restraint, by baseline characteristics and severity of illness, may affect outcomes and may have introduced further bias. However, as a scoping review, it was thought important to explore all avenues of investigating outcomes in ETN. Although this review included studies with participants diagnosed with AAN, evidence related to AAN could not be differentiated from that associated with AN. Most participants were female, with only one study conducted in an exclusively male population. Therefore, results may not be generalisable to male sufferers. Differentiation of outcomes related to the age of participants could not be made. Furthermore, most studies came from Europe, North America, and Australia, and results may not be generalisable to populations from other continents.

### 4.6. Future Research Recommendations

**Figure 2:**
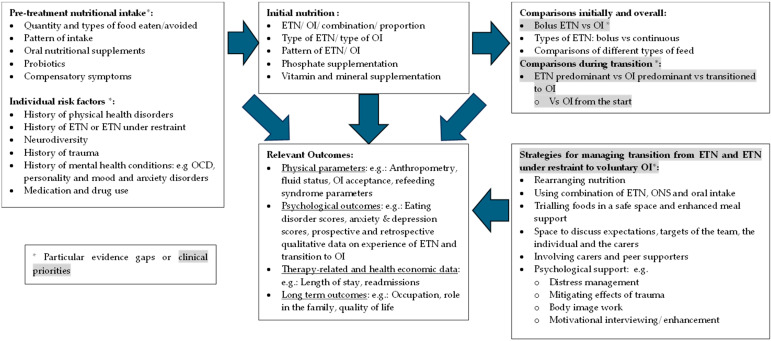
Examples of future research recommendations in the field of enteral nutrition for underweight people with eating disorders.

Research suggestions in areas that have gaps in the evidence base are presented in Figure 2. Populations in which more research would be beneficial would be those presenting with AAN. Furthermore, those with AN or AAN and co-morbidities or conditions that have the potential to prolong ETN use; for example, personality disorders, mood and anxiety disorders, and autism, require more research. Comparing those with a recent onset eating disorder with those who have had a long-standing eating disorder would be important. Additionally, research is needed in AN and AAN in male and gender-diverse populations. Furthermore, research comparing outcomes based on age categories would support differentiating strategies in paediatric and adult populations.

Interventions that would benefit from more evidence include the use of ETN as bolus feeds and bolus replacement feeds, as this method is commonly used in eating disorders treatment, and most of the current evidence relates to continuous ETN. The use of propensity scoring, such as used in Martini et al. (2024) [44], matching characteristics, including severity, of participants in the ETN and OI naturalistic cohorts, may support making comparisons more meaningful.

More details on the methods of ETN feeding procedures followed, including the proportion of nutrition through ETN and OI and relating these with pre-treatment OI, would be important. Additionally, methods of transition from ETN and the outcomes need to be explored. Psychological support strategies, to reduce distress and ambivalence about recovery, with a personalised approach, need further research.

Comparing outcomes between ETN-predominant, OI predominant, and OI-only phases during the nutritional transition from ETN to OI, would enhance knowledge and guide supportive strategies. Additionally, connecting quantitative and qualitative data within individuals in a study to co-produce care pathways may be helpful. Furthermore, exploring a wider range of potential recovery-related biomarkers, such as the gut microbiome, metabolic, and immunological parameters, may be of value.

## 5. Conclusions

ETN is a viable option for improvement in physical health when this route of nutrition is needed. The evidence so far shows that it can support weight restoration without increasing the risk of RFS or the risk of readmissions. ETN may increase the length of stay, and the reasons for this need to be explored. The evidence so far indicates an overall improvement in eating disorder related scores with nutritional treatment; however, more research on psychological outcomes and exploring strategies to support recovery in the context of ETN is required. More research is needed in exploring methods of transition that are effective and supportive of service users' ability to accept responsibility for their nutrition. Research into mitigating trauma associated with NG under restraint is also needed.

## Data Availability

No new data were created or analysed in this study. Data sharing is not applicable to this article.

## Supplementary Materials

The following supporting information can be downloaded at: https://www.mdpi.com/article/10.3390/nu17030425/s1, Table S1: Summary of initial nutritional protocols, Table S2: Summary of psychological outcomes and Table S3: Summary of qualitative data.

## References

[1] American Psychiatric Association (2013). Diagnostic and Statistical Manual of Mental Disorders.

[2] Himmerich H., Hotopf M., Shetty H., Schmidt U., Treasure J., Hayes R.D., Stewart R., Chang C.K. (2019). Psychiatric comorbidity as a risk factor for mortality in people with anorexia nervosa. Eur. Arch. Psychiatry Clin. Neurosci..

[3] Brennan C., Illingworth S., Cini E., Bhakta D. (2023). Medical instability in typical and atypical adolescent anorexia nervosa: A systematic review and meta-analysis. J. Eat. Disord..

[4] National Institute for Health and Care Excellence (2017). Eating Disorders: Recognition and Treatment.

[5] Royal College of Psychiatrists Medical Emergencies in Eating Disorders: Guidance on Recognition and Management. https://www.rcpsych.ac.uk/docs/default-source/improving-care/better-mh-policy/college-reports/college-report-cr233-medical-emergencies-in-eating-disorders-(meed)-guidance.pdf?sfvrsn=2d327483_63.

[6] Agostino H., Erdstein J., Di Meglio G. (2013). Shifting Paradigms: Continuous Nasogastric Feeding With High Caloric Intakes in Anorexia Nervosa. J. Adolesc. Health.

[7] Watters A., Gibson D., Dee E., Mascolo M., Mehler P.S. (2020). Superior mesenteric artery syndrome in severe anorexia nervosa: A case series. Clin. Case Rep..

[8] Reber E., Friedli N., Vasiloglou M.F., Schuetz P., Stanga Z. (2019). Management of Refeeding Syndrome in Medical Inpatients. J. Clin. Med..

[9] National Institute for Health and Care Excellence (2006). Nutrition Support for Adults: Oral Nutrition Support, Enteral Tube Feeding and Parenteral Nutrition.

[10] Parker E.K., Flood V., Halaki M., Wearne C., Anderson G., Gomes L., Clarke S., Wilson F., Russell J., Frig E. (2021). A standard enteral formula versus an iso-caloric lower carbohydrate/high fat enteral formula in the hospital management of adolescent and young adults admitted with anorexia nervosa: A randomised controlled trial. J. Eat. Disord..

[11] Kohn M.R., Madden S., Clarke S.D. (2011). Refeeding in anorexia nervosa: Increased safety and efficiency through understanding the pathophysiology of protein calorie malnutrition. Curr. Opin. Pediatr..

[12] Bendall C., Taylor N.F. (2023). The effect of oral refeeding compared with nasogastric refeeding on the quality of care for patients hospitalised with an eating disorder: A systematic review. Nutr. Diet. J. Dietit. Assoc. Aust..

[13] Rizzo S.M., Douglas J.W., Lawrence J.C. (2019). Enteral Nutrition via Nasogastric Tube for Refeeding Patients With Anorexia Nervosa: A Systematic Review. Nutr. Clin. Pract..

[14] Tricco A.C., Lillie E., Zarin W., O'Brien K.K., Colquhoun H., Levac D., Moher D., Peters M.D.J., Horsley T., Weeks L. (2018). PRISMA Extension for Scoping Reviews (PRISMA-ScR): Checklist and Explanation. Ann. Intern. Med..

[15] Ouzzani M., Hammady H., Fedorowicz Z., Elmagarmid A. (2016). Rayyan—A web and mobile app for systematic reviews. Syst. Rev..

[16] Moola S.M.Z., Tufanaru C., Aromataris E., Sears K., Sfetcu R., Currie M., Qureshi R., Mattis P., Lisy K., Mu P.-F., Aromataris E.M.Z. (2020). Systematic reviews of etiology and risk. JBI Manual for Evidence Synthesis.

[17] Barker T.H., Habibi N., Aromataris E., Stone J.C., Leonardi-Bee J., Sears K., Hasanoff S., Klugar M., Tufanaru C., Moola S. (2024). The revised JBI critical appraisal tool for the assessment of risk of bias for quasi-experimental studies. JBI Evid. Synth..

[18] Rigaud D., Brondel L., Poupard A.T., Talonneau I., Brun J.M. (2007). A randomized trial on the efficacy of a 2-month tube feeding regimen in anorexia nervosa: A 1-year follow-up study. Clin. Nutr..

[19] Rigaud D.J., Brayer V., Roblot A., Brindisi M.C., Verges B. (2011). Efficacy of tube feeding in binge-eating/vomiting patients: A 2-month randomized trial with 1-year follow-up. J. Parenter. Enter. Nutr..

[20] Madden S., Miskovic-Wheatley J., Wallis A., Kohn M., Lock J., Le Grange D., Jo B., Clarke S., Rhodes P., Hay P. (2015). A randomized controlled trial of in-patient treatment for anorexia nervosa in medically unstable adolescents. Psychol. Med..

[21] Matthews-Rensch K., Young A., Cutmore C., Davis A., Jeffrey S., Patterson S. (2023). Acceptability of using a nasogastric refeeding protocol with adult patients with medically unstable eating disorders. J. Eval. Clin. Pract..

[22] Fuller S.J., Tan J.C.T., Nicholls D. (2023). Nasogastric tube feeding under restraint: Understanding the impact and improving care. BJPsych Bull..

[23] Fuller S.J., Tan J.C.T., Nicholls D. (2024). The importance of individualised care, good communication and trust for reducing nasogastric tube feeding under physical restraint: Qualitative multi-informant study. BJPsych Open.

[24] Silber T.J., Robb A.S., Orrell-Valente J.K., Ellis N., Valadez-Meltzer A., Dadson M.J. (2004). Nocturnal nasogastric refeeding for hospitalized adolescent boys with anorexia nervosa. J. Dev. Behav. Pediatr. JDBP.

[25] Kells M., Gregas M., Wolfe B.E., Garber A.K., Kelly-Weeder S. (2022). Factors associated with refeeding hypophosphatemia in adolescents and young adults hospitalized with anorexia nervosa. Nutr. Clin. Pract..

[26] Pruccoli J., Barbieri E., Visconti C., Pranzetti B., Pettenuzzo I., Moscano F., Malaspina E., Marino M., Valeriani B., Parmeggiani A. (2024). Refeeding syndrome and psychopharmacological interventions in children and adolescents with Anorexia Nervosa: A focus on olanzapine-related modifications of electrolyte balance. Eur. J. Pediatr..

[27] Kezelman S., Rhodes P., Hunt C., Anderson G., Clarke S., Crosby R.D., Touyz S. (2016). Adolescent patients’ perspectives on rapid-refeeding: A prospective qualitative study of an inpatient population. Adv. Eat. Disord..

[28] Mac Donald B., Gustafsson S.A., Bulik C.M., Clausen L. (2023). Living and leaving a life of coercion: A qualitative interview study of patients with anorexia nervosa and multiple involuntary treatment events. J. Eat. Disord..

[29] Halse C., Boughtwood D., Clarke S., Honey A., Kohn M., Madden S. (2005). Illuminating multiple perspectives: Meanings of nasogastric feeding in anorexia nervosa. Eur. Eat. Disord. Rev..

[30] Madden S., Miskovic-Wheatley J., Clarke S., Touyz S., Hay P., Kohn M.R. (2015). Outcomes of a rapid refeeding protocol in Adolescent Anorexia Nervosa. J. Eat. Disord..

[31] Kezelman S., Crosby R.D., Rhodes P., Hunt C., Anderson G., Clarke S., Touyz S. (2018). Anorexia nervosa, anxiety, and the clinical implications of rapid refeeding. Front. Psychol..

[32] Paccagnella A., Mauri A., Baruffi C., Berto R., Zago R., Marcon M.L., Pizzolato D., Fontana F., Rizzo L., Bisetto M. (2006). Application criteria of enteral nutrition in patients with anorexia nervosa: Correlation between clinical and psychological data in a “lifesaving” treatment. JPEN J. Parenter. Enter. Nutr..

[33] Hanachi M., Melchior J.C., Crenn P. (2013). Hypertransaminasemia in severely malnourished adult anorexia nervosa patients: Risk factors and evolution under enteral nutrition. Clin. Nutr..

[34] Rigaud D., Tallonneau I., Brindisi M.C., Verges B. (2012). Prognosis in 41 severely malnourished anorexia nervosa patients. Clin. Nutr..

[35] Minano Garrido E., Di Lodovico L., Dicembre M., Duquesnoy M., Ohanyan H., Melchior J.C., Hanachi M. (2021). Evaluation of muscle-skeletal strength and peak expiratory flow in severely malnourished inpatients with anorexia nervosa: A pilot study. Nutrition.

[36] Robb A.S., Silber T.J., Orrell-Valente J.K., Valadez-Meltzer A., Ellis N., Dadson M.J., Chatoor I. (2002). Supplemental nocturnal nasogastric refeeding for better short-term outcome in hospitalized adolescent girls with anorexia nervosa. Am. J. Psychiatry.

[37] Born C., de la Fontaine L., Winter B., Muller N., Schaub A., Frustuck C., Schule C., Voderholzer U., Cuntz U., Falkai P. (2015). First results of a refeeding program in a psychiatric intensive care unit for patients with extreme anorexia nervosa. BMC Psychiatry.

[38] Gentile M.G. (2012). Enteral nutrition for feeding severely underfed patients with anorexia nervosa. Nutrients.

[39] Murciano D., Rigaud D., Pingleton S., Armengaud M.H., Melchior J.C., Aubier M. (1994). Diaphragmatic function in severely malnourished patients with anorexia nervosa: Effects of renutrition. Am. J. Respir. Crit. Care Med..

[40] Rigaud D., Boulier A., Tallonneau I., Brindisi M.C., Rozen R. (2010). Body fluid retention and body weight change in anorexia nervosa patients during refeeding. Clin. Nutr..

[41] Zuercher J.N., Cumella E.J., Woods B.K., Eberly M., Carr J.K. (2003). Efficacy of voluntary nasogastric tube feeding in female inpatients with anorexia nervosa. J. Parenter. Enter. Nutr..

[42] Blikshavn T., Halvorsen I., Ro O. (2020). Physical restraint during inpatient treatment of adolescent anorexia nervosa: Frequency, clinical correlates, and associations with outcome at five-year follow-up. J. Eat. Disord..

[43] Marchili M.R., Diamanti A., Zanna V., Spina G., Mascolo C., Roversi M., Guarnieri B., Mirra G., Testa G., Raucci U. (2023). Early Naso-Gastric Feeding and Outcomes of Anorexia Nervosa Patients. Nutrients.

[44] Martini M., Longo P., Di Benedetto C., Delsedime N., Panero M., Abbate-Daga G., Toppino F. (2024). Nasogastric Tube Feeding in Anorexia Nervosa: A Propensity Score-Matched Analysis on Clinical Efficacy and Treatment Satisfaction. Nutrients.

[45] Trovato C.M., Capriati T., Bolasco G., Campana C., Papa V., Mazzoli B., Zanna V., Marchili M.R., Basso M.S., Maggiore G. (2022). Five-Year Inpatient Management of Teenagers With Anorexia Nervosa: Focus on Nutritional Issues. J. Pediatr. Gastroenterol. Nutr..

[46] Braude M.R., Con D., Clayton-Chubb D., Nandurkar R., Chua L.E., Newnham E.D. (2020). Acute medical stabilisation of adults with anorexia nervosa: Experience of a defined interdisciplinary model of care. Intern. Med. J..

[47] Gentile M.G., Manna G.M., Ciceri R., Rodeschini E. (2008). Efficacy of inpatient treatment in severely malnourished anorexia nervosa patients. Eat. Weight. Disord..

[48] Rigaud D., Pennacchio H., Bizeul C., Reveillard V., Verges B. (2011). Outcome in AN adult patients: A 13-year follow-up in 484 patients. Diabetes Metab..

[49] Bufano G., Bellini C., Cervellin G., Coscelli C. (1990). Enteral nutrition in anorexia nervosa. JPEN J. Parenter. Enteral. Nutr..

[50] Nehring I., Kewitz K., von Kries R., Thyen U. (2014). Long-term effects of enteral feeding on growth and mental health in adolescents with anorexia nervosa--results of a retrospective German cohort study. Eur. J. Clin. Nutr..

[51] Pruccoli J., Pelusi M., Romagnoli G., Malaspina E., Moscano F., Parmeggiani A. (2021). Timing of psychopharmacological and nutritional interventions in the inpatient treatment of anorexia nervosa: An observational study. Brain Sci..

[52] Pruccoli J., Pettenuzzo I., Parmeggiani A. (2022). Treatment response in children and adolescents with anorexia nervosa: A naturalistic, case-control study. Eat. Weight. Disord. EWD.

[53] Hale M.D., Logomarsino J.V. (2019). The use of enteral nutrition in the treatment of eating disorders: A systematic review. Eat. Weight. Disord..

[54] Hindley K., Fenton C., McIntosh J. (2021). A systematic review of enteral feeding by nasogastric tube in young people with eating disorders. J. Eat. Disord..

[55] Fuller S.J., Tan J., Nicholls D. (2023). Decision-making and best practice when nasogastric tube feeding under restraint: Multi-informant qualitative study. BJPsych Open.

[56] Conti J.E., Joyce C., Hay P., Meade T. (2020). “Finding my own identity”: A qualitative metasynthesis of adult anorexia nervosa treatment experiences. BMC Psychol..

[57] Morgan K., Cutmore C., Matthews-Rensch K. (2023). Adding mini meals to a nasogastric refeeding protocol for patients with eating disorders can be achieved on general hospital wards. J. Hum. Nutr. Diet. Off. J. Br. Diet. Assoc..

[58] Couturier J., Mahmood A. (2009). Meal support therapy reduces the use of nasogastric feeding for adolescents hospitalized with anorexia nervosa. Eat. Disord..

[59] Krom H., de Winter J.P., Kindermann A. (2017). Development, prevention, and treatment of feeding tube dependency. Eur. J. Pediatr..

