# Supplementary material for: Enteral Tube Nutrition in Anorexia Nervosa and Atypical Anorexia Nervosa and Outcomes: A Systematic Scoping Review
Source: Nutrients. 2025 Jan 24;17(3):425. doi: 10.3390/nu17030425 (PMC11820346; doi:10.3390/nu17030425)
Supplement: Supplementary file 1 [file nutrients-17-00425-s001.zip › Table S1 initial nutritional protocols table 17.01.25.pdf]

Table S1: Summary of initial nutritional protocols

**Author, year**	**Initial nutritional protocols**					
	Cohort (s), specifics, n	*Initial nutrition route and overview*	*Energy prescribed (*kcal or **kcal/kg/d***)*<br>*Mean ±s.d*<br>*Median (range)*	*Feed type: Prophylactic PO4: Y/N/NR*	*Feed admin*	*OI/ ONS*
Agostino et al., 2013. [6]	1. NG gp: n=31	NG only (max 7 days) initially.<br>OI introduced: -once overnight medical stabilisation (med. stab.)<br>+ max. energy via NG<br>or 7 days NG completed	Day 1: 1500-1800. (actual intake: 1617 ± 276*)<br>Day 2 onwards: ↑ by 200 until target reached	Polymeric feed, 44% CHO<br><br>PO4: Y	Continuous feed.<br>NG only for max 7 days	OI introduced as part of transition with OI ↑ and NG ↓ over 3 days
	2. OI gp: n=134	OI intake only	Day 1: 1000-1200. (actual intake: 1069 ± 212*)<br>Day 2 onwards: ↑ by 150 until target reached	NA<br><br>PO4: NR	NA	3 meals + 3 snacks.
Blikshavn et al., 2020 [42]	1. NG under restraint (NGR): n=8	NG under restraint when needed	NR	NR	NR<br><br>Average duration of NGR: 19.4 ± 9.2 minutes	1st line OI, then ONS, then, if needed, NG
	2. No NGR: n=30	No NGR.<br>Whether received feed via NG not under restraint - NR	NR	NR	NR	1st line OI, then ONS, then, if needed, NG
Born et al., 2015 [37]	1. PEG gp: n=57	PEG nutrition + OI	- Initial energy NR<br>- Individualised prescription	'Hypercaloric' feed<br><br>PO4: NR	NR	OI from the start

	2. No PEG gp: n=11	OI or NG or both.	- Initial energy NR<br>- Individualised prescription.	NR	NR	OI from the start
Braude et al., 2020 [46]	1. NG gp: n=27	NG – if unable to comply with OI, too unwell or high risk	NR	NR	NG duration: median (IQR): 6 (2-12) days	NR
	2. OI gp: n=68	OI - 1st line.	NR	NA<br><br>PO4: NR	NA	NR
Bufano et al., 1990 [49]	1. NG gp: n=9	NG only initially	Day 1: 25% estimated req.<br>Day 2: 50% req.<br>Day 3: 75% req.<br>Day 4 onwards: 100% req.<br>*Average prescription*: 2311 ± 607	Polymeric feed, 44% CHO, 36% F, 19.7% P<br><br>PO4: N	Continuous feed over 14 hours<br>Duration of NG: 21 ± 14 days	NA
Gentile, 2012 [38]	1. NG gp: n=10	NG + OI + glucose	Day 1: NG: 450 + OI 408 + glucose 200 = 1199<br>Day 15: NG 837.5 + OI 1204 + glucose 400 = 2508	Polymeric feed, 1.7-2 kcal/ml<br><br>PO4: Y	Continuous over 24 hrs	OI from the start.<br>Personalised meal plan
Gentile et al., 2008 [47]	1. NG gp: n=32	NG under life threatening conditions + OI from the start	No specifics.<br>Average NG prescription: 1375 ± 211	NR	NR<br><br>NG duration: 4.4 ± 2.5 months.	OI from the start.<br>Personalised meal plan
	2. OI gp: n= 67	OI + /- ONS	NA	NA<br><br>PO4: NR	NA	Personalised meal plan
Hanachi et al., 2013 [33]	1. ↑ AST/ALT gp w/ NG: n=54	ETN only initially – type of tube NR	Day 1: IV fluids + vitamin & mineral supplement.<br>Day 2: **10kcal/kg/d**<br>Day 3 onwards: slow ↑to **30kcal/kg/d**<br>Week 1 average prescription: 441 ±141	Polymeric, isotonic feed<br><br>PO4: Y	Continuous<br>Duration: 4 weeks	OI introduced later
	2. AST/ALT ↔ gp w/NG: n=72	ETN only initially – type of tube NR	Days 1 onwards as above.<br>Week 1 average prescription: 630 ± 178	Polymeric, isotonic feed	Continuous.<br>Duration: 4 weeks	OI introduced later

				PO4: Y		
Kells et al., 2022 [25]	1. NG gp: n=44	NG if OI/ONS not accepted	1000-3000kcal. ↑by 250 in steps. Average initial prescription: 1714 ± 324.5	Feed type: NR<br><br>PO4: Y	Bolus feed by the bedside	OI 1st line.<br>Pattern: meals + snacks. ONS as replacement if OI not accepted.<br>NG if OI/ ONS not accepted.
	2. OI gp: n=256	OI ± ONS	Prescription as above	NA<br><br>PO4: Y	NA	Pattern and ONS replacement as above
Kezelman et al., 2018 [31]	1. NG gp: n=31	NG only initially.<br>OI introduced when med. stab.	Day 1: 2400 Continued until OI added. then<br>↑ to max. 3800.	Feed type: NR<br><br>PO4: Y	Day 1: continuous 24-hour feed until OI added. From then on continuous nocturnal feed with daytime OI	OI added when med. Stab.<br><br>Pattern: 3 meals + 3 snacks.<br><br>5 staged meal plans 1800 – 3800kcal.
Madden et al., 2015a [30]	1. NG gp: n=78	NG only initially. OI introduced with med. stab. NG continued until Day 14 at least.	Day 0-3: 2400.<br>Days 1-7 (when med stab.): Via NG: 1000. +<br>Via OI: 1500-1800	1kcal/ml feed. <50% energy via CHO, 30% via F.<br><br>PO4: Y	Day 0-3 (depending on med. stab.) – continuous, 100ml/hr x 24 hrs.<br>Days 1-7 (when med. stab.): nocturnal continuous, 100ml/hr x 10 hrs.<br>Daytime OI	OI added when med. stab.<br><br>OI: meal plans with staged energy prescriptions: 1500-3000
Madden et al., 2015b [20]	1. Arm aiming for med stab. before hospital discharge (d/c) (MS gp): n=41	NG only initially (24-72 hrs). Transition to nocturnal NG + daytime OI with daytime med stab.	Initial: 2400.<br>↑ to 3000 in steps.	NR<br><br>PO4: NR	Continuous feed. 24 hr initially, then nocturnal when daytime med. stab.	OI: meal plans – no specifics
	2. Arm aiming for weight restoration before d/c	As above	As above	As above<br><br>PO4: NR	As above	As above

	(WR gp): n=41					
Marchili et al., 2023 [43]	1. NG gp: n=101	NG started if <30% of OI + ONS managed	NR	1.5kcal/ml.<br><br>PO4: NR	Continuous feed.<br>Varied timings: nocturnal, daytime only or all day.<br>Day when NG started, median (IQR): 5 (0-17) days.<br>Duration of NG: 21 ± 13 days.	OI pattern: NR
	2. OI gp: n=214	OI 1st line. If <70% of OI managed, ONS added	NR	NA<br><br>PO4: NR	NA	OI pattern: NR
Martini et al., 2024 [44]	1. NG gp: n=97	NG supplementation if difficulties with OI. OI from the start	NR	NR	Continuous daytime feed	OI from the start. Meals 5 times/day
	2. OI gp: n=97	OI first line.	NR	NA	NA	Meals 5 times/day
Minano Garrido et al., 2021 [35]	1. ETN gp: n=23	ETN only initially - type of tube NR<br>OI introduced subsequently.	Days 1-2: **10kcal/kg/d**.<br>Days 3 onwards: ↑ to **50kcal/kg/d** slowly.	Polymeric<br><br>PO4: Y	Not specified.<br>Duration of study with ETN: 5 weeks.	OI introduced with weight ↑<br><br>Pattern – not specified
Murciano et al., 1994 [39]	1. NG gp: n=15	Days 0-5: 'spontaneous nutrition'<br>Day 5 onwards: NG started. OI not specified.	Week 1 average prescription: 1824 ± 399	Polymeric, 1kcal/ml, 48% CHO, 36% F, 16% P.<br><br>PO4: NR	Continuous.<br>NG duration: at least 6 weeks.	OI: unclear if used beyond day 5.
Nehring et al., 2014 [50]	1. NG gp: n=71	NG in at least 1 admission	NR	NR	NR	NR
	2. OI gp: n=137	OI only through all admissions	NR	NR	NR	NR

Paccagnella et al. ,2006 [32]	1. NG gp: n=24	Initially NG + IV fluids<br>Day 3-4 onwards OI introduced.	Phase 1 (initial phase): <1000.<br>Phase 2: slight ↑in prescription.<br>Days 1-2: via NG: 630. Via OI: 0.<br>Days 10-12: NG: 1005 + OI: 450 = total: 1455	Phase 1: Polymeric, 1kcal/ml, CHO 44%, F 36%, P 19.7%.<br><br>PO4: NR	Continuous over 24 hrs.<br><br>Overall NG duration: 20.7 ± 7.1 days	OI: Introduced on days 3-4<br><br>Mediterranean diet, spontaneous intake
Parker et al., 2021 [10]	1. Low CHO feed gp: n=15	NG only initially<br>Once med. stab. OI introduced.	Day 1: via NG: 1260-1890 (based on electrolyte levels).<br>Day 2 onwards until med stab: via NG: 2500<br>When med. Stab.: nocturnal NG and daytime OI.<br>By week 1: 3350 (3188, 3350)	Polymeric feed, 1.5kcal/ml, 28% CHO, 56% F, 17% P<br><br>PO4: only if levels <=1mmol/L prior to refeeding	Continuous feed.<br>Day 1: 35ml/hr x 12 hrs. if electrolytes within range, rate ↑ to 70ml/hr x 12 hrs. (if electrolytes abnormal, rate stayed at 35ml/hr).<br>Day 2 onwards until med stab: 70ml/hr x 24hrs.<br>When med stab., nocturnal feed.<br>Replacement feed, if necessary, also added.	OI introduced when med. stab (average 2 days).<br>Staged plans: 1800-3800kcal.<br>Content: CHO 47-57%, F 30-38%, P 13 - 15%
	2. Standard CHO feed gp: n=11	NG only initially<br>Once med. stab. OI introduced.	Prescription like above gp.<br>By week 1: 3325 (2844, 3350)	Polymeric feed, 1.5kcal/ml, 54% CHO, 29% F, 17% P<br><br>PO4: only if levels <=1mmol/L prior to refeeding	As above	As above gp. No difference in OI CHO content between gps.
Pruccoli et al., 2021 [51]	1. Early AAP + Early NG: n= 18	NG within 7 days of admission (early NG). Unclear if OI also used with NG.	NR	NR	At least 7 days NG nutrition	NR
	2. Early AAP + late NG: n=12	NG started >7 days after admission (late NG). OI as above	NR	NR	As above	NR

	3. Late AAP + early NG: n=20	Early NG. OI as above	NR	NR	As above	NR
	4. Late AAP + late NG: n=18	Late NG. OI as above	NR	NR	As above	NR
	5. NG without AAP: n=11	Early NG: n=5, late NG: n=6. OI as above	NR	NR	As above	NR
Pruccoli et al., 2022 [52]	1. NG gp: n=33	Group receiving some NG during their admission	NR	NR	NR	NR
	2. OI gp: n=43	OI only	NR	NR	NR	NR
Pruccoli et al., 2024 [26]	1. NG gp: n=44	NG during admission if severely malnourished or struggling with OI	Starting at 15-20kcal/kg/d in 1st 24 hrs. increase every 2-3 days. High risk: 5-10kcal/kg/d, max of 20kcal/kg/d in week 1	NR	NR	NG supplementing OI
	2. other (mainly OI): n=69	OI mainly. n=1 PN specified under RFS gp. PN given if NG or OI impractical or insufficient	As above	N/A	N/A	Balanced meals through the day, ONS if needed.
Rigaud et al., 2010 [40]	1. Low sodium OI gp: n=176	NG and low sodium OI from the start	Week 1:<br>1583 ± 207 – based on estimated maintenance req.	Polymeric, 1kcal/ml, 0.6g NaCl/L<br><br>PO4: NR	NR	OI from the start:<br>Pattern: 3 meals + 1-2 snacks.<br>Sodium content of OI: 5g/2000kcal/d
	2. Std sodium OI gp: n=42	NG and standard sodium OI from the start	Week 1:<br>1638 ± 234- based on estimated maintenance req.	polymeric, 1kcal/ml, 0.6g NaCl/L<br><br>PO4: NR	NR	OI from the start:<br>Pattern: 3 meals + 1-2 snacks.<br>Sodium content: 10-12g/d

Rigaud et al., 2007 [18]	1. NG gp (intervention arm): n=41	NG + OI from the start	Target energy prescription: for weight gain of 1kg/week.<br>If BMI <12.5: Week 1: **<30kcal/kg/d**.<br>Week 2: **<35kcal/kg/d**	Polymeric, no fibre<br><br>PO4: Y	Continuous, during daytime, 90-150ml/hr.<br>NG titrated based on OI to achieve 1kg/week weight gain.	Pattern: meals and snacks.<br>Sodium intake: <4-5g/d.<br>OI energy: Day 1: AN-R: 850 ± 124. AN-BP: 1234 ± 321<br>Day 15: AN-R: 1876 ± 211. AN-BP: 1785 ± 187
	2. OI gp (control arm): n=40	OI only	As per intervention arm	NA<br><br>PO4: Y	NA	Pattern: meals and snacks.<br>Sodium intake: <4-5g/d.<br>No ONS prescribed.<br>OI energy: Day 1: AN-R: 942 ± 175. AN-BP: 1098 ± 279.<br>Day 15: AN-R: 1702 ± 193. AN-BP: 1613 ± 237
Rigaud et al., 2011a [48]	1. NG gp: n=262	NG if unable to gain weight with OI only or if several BP episodes.	NR	NR<br><br>PO4: NR	NG for >2months.	OI from the start<br><br>Details NR
	2. OI gp: n=222	OI	NR	NA<br><br>PO4: NR	NA	As above
Rigaud et al., 2012 [34]	1. NG gp: n=41	NG only initially. OI introduced from Day 11-21	Day 1: average intake 309 ± 18 via IV glucose+ fluids.<br>Day 2: **25kcal/kg/d** via NG + IV lipids.<br>Day 3: average intake: 754 ± 62<br>Days 4-5: **35kcal/kg/d** via NG and IV lipids.<br>Day 6-10: 35-**40kcal/kg/d** via NG, IV lipids stopped. day 8-10: 1105 ± 118 via NG.	Polymeric<br><br>PO4: Y	NR	Days 11-21: OI introduced as small meals (300-400kcal).<br>Sodium <4g/d.<br>Total energy including NG and OI: 45kcal/kg/d.<br>From week 4: aim for weight gain of 0.7-1kg/week. OI: <6g sodium/d until BMI 15 reached.
Rigaud et al., 2011b [19]	1. NG + CBT gp: n=52 (AN subgp: n=19)	NG only nutrition initially for 3 weeks. OI introduced from week 4	NR	Polymeric<br><br>PO4: NR	Initial NG only duration: 3 weeks. NG continued for 2 months.	OI introduced in week 4 only<br>Pattern: 3 meals + snack.<br>Stepwise introduction: breakfast, then lunch, then dinner.

	2. OI + CBT gp: n=51 (AN subgp n=17)	OI only	NR	NA<br><br>PO4: NR	NA	Pattern: 3 meals + snack
Robb et al., 2002 [36]	1. NG gp: n=52	NG + OI from the start	Energy prescription: to achieve target weight gain of 1-2kg/week.<br>OI:NG: 2:1	1.5kcal/ml feed<br><br>PO4: N	Continuous nocturnal.<br>Night 1: 40ml/hr x 4 + 60ml/hr x 4 = 400ml (600kcal).<br>Night 2: 80ml/hr x 4 + 100ml/hr x 4 = 720ml (1080kcal).<br>Night 3 onwards: 100ml/hr x 8 = 800ml (1200kcal)	Day time OI.<br>Pattern: 3 meals + 2 snacks. ONS as needed.
	2. OI gp: n=48	OI only	OI gradually ↑. Target weight gain of 1-2kg/week	NA<br><br>PO4: N	NA	Pattern: 3 meals + 2 snacks. ONS as needed.<br>Further into admission – more responsibility with meal choices given.
Silber et al., 2004 [24]	1. NG gp: n=6	NG + OI from the start	Gradual ↑ to prevent RFS.	NR<br><br>PO4: NR	Nocturnal NG.<br>OI not replaced via NG	Day time OI
	2. OI gp: n=8	OI only	Gradual ↑ to prevent RFS.	NA<br><br>PO4: NR	NA	NR
Trovato et al., 2022 [45]	1. NG + ONS + OI gp: n=51	If OI + ONS <30% of req., NG added within 1st week of admission	At least 40kcal/kg/d	Polymeric, 1kcal/ml feed<br><br>PO4: NR	Continuous.<br>Initially 35ml/hr ↑gradually over 9 days.<br>Max 1500ml (1500kcal)	OI from the start.<br>Aiming for 40kcal/kg/d.<br>CHO 40-65%. P: 10-35%. F: 20-35%<br>ONS (1.5kcal/ml) added as per ONS protocol
	2. ONS + OI gp: n=63	If OI <70% of req. ONS added.	At least **40kcal/kg/d**	NA<br><br>PO4: NR	NA	As above
	3. OI gp: n=66	OI 1st line.	At least **40kcal/kg/d**	NA<br><br>PO4: NR	NA	As above

	4. NG+ OI gp: n=6	As per NG protocol, but ONS not used.	At least **40kcal/kg/d**	Polymeric, 1kcal/ml feed<br><br>PO4: NR	Continuous.<br>Initially 35ml/hr<br>↑gradually over 9 days.<br>Max 1500ml (1500kcal)	As above
Zuercher et al., 2003 [41]	1. NG gp: n=155	NG + OI from the start	NR<br><br>Target weight gain: >1kg/week.	Polymeric, 1.2-1.5kcal/ml feed.<br><br>PO4: NR	Continuous, nocturnal, over 4-8 hrs.<br>Typical rate: 50ml/hr x 4 hrs.<br>↑by 300kcal/3days via rate or duration till target weight gain reached.<br><br>NG duration overall: 36 (21) days.	OI from the start.<br>Pattern: 3 meals + snacks.
	2. OI gp: n=226	OI only.<br><br>NG recommended but not accepted	Target weight gain: >1kg/week	NA<br><br>PO4: NR	NA	Pattern: 3 meals + snacks.
