# Supplementary material for: Enteral Tube Nutrition in Anorexia Nervosa and Atypical Anorexia Nervosa and Outcomes: A Systematic Scoping Review
Source: Nutrients. 2025 Jan 24;17(3):425. doi: 10.3390/nu17030425 (PMC11820346; doi:10.3390/nu17030425)
Supplement: Supplementary file 1 [file nutrients-17-00425-s001.zip › Table S2 psychological outcomes table 17.01.25.pdf]

Table S2: Studies reporting psychological outcomes

Author, year	Cohort (s)(n).	Psychological outcomes
Blikshavn et al., 2020 [42]	1. NG-R (n=8)<br>2. no NG-R (n=30)	1. vs 2.: At 5-year FU: EDE-Q worse trend n.s.<br>1. n=6/8 → highest number of restraints during admission →all had ED diagnosis at FU.
Kezelman et al., 2018 [31]	1. NG (n=31)	BAI, BDI, STAI-Y1 ↓ over time during admission* →not associated with BMI ↑.<br>Anti-psychotic medication associated with ↓ depressive symptoms*.<br>STAI-Y2 and ACQ changes over time: n.s.<br>EDE-Q -global, restraint & eating concerns ↓ over time*.<br>Weight and shape concerns changes over time – n.s.
Madden et al., 2015b** [20]	1. MS gp w/NG (n=41)<br>2. WR gp w/NG (n=41)	1. vs 2.:<br>EDE- Q change: - 1.53 ± 1.48 vs – 1.35 ± 1.58 n.s<br>Full remission at 12- month FU (EDE- Q within 1 SD of mean): 30% vs 32.5%. n.s.
Martini et al., 2024 [44]	1. NG (n=97)<br>2. OI (n=97)	1. vs 2.: higher bond between patient and clinician in 1. *<br>EDE-Q total score reduction: 0.58 (1.43) vs 0.81 (1.30) n.s.
Nehring et al., 2014 [50]	1. NG (n=71)<br>2. OI (n=137)	1. vs 2.: At FU (mean 6 yrs)<br>Persistence of AN diagnosis & occurrence of other mental health conditions – n.s.
Paccagnella et al. ,2006 [32]	1. NG (n=24)	In subgp aged 15-20 yrs (n=8): late refeeding vs early refeeding: 'treatment willingness score' (lower score = accepting of OI) improved*.<br>Affecting this score:<br>In early refeeding: → anxiety*, interoceptive awareness*<br>Late refeeding: → obsessive compulsive symptoms*<br>FU → anxiety* and interpersonal distrust*
Pruccoli et al., 2022 [52]	1. NG (n=33)<br>2. OI (n=43)	Good outcome gp (completed initial admission, available for FU at 6 mon. and %mBMI >70% at FU) vs treatment resistant gp: NG use in admission: n=23 (50%) vs n=10 (33.3%)*<br>At admission:<br>EDI 3 EDRC score: 65.6±23.2 vs 78.4±17.9*<br>BDI II: 25.6±13.3 vs 31.3±9.0 n.s.
Rigaud et al., 2007** [18]	1. NG (intervention gp, n=41)<br>2. OI (control gp, n=40)	1. vs 2.:<br>Introduction of new foods similar.<br>BP episodes/ week lower*<br>EDI score ↓ from admission to 1 yr FU in both groups*, n.s between gps
Rigaud et al, 2011b** [19]	1. NG + CBT (n=52, AN subgp: n=19)<br>2. OI + CBT (n=51, AN subgp: n=17)	1. vs 2.:<br>QoL improvement at 8 weeks higher*, similar improvement at 1 yr FU*<br>EDI score improved* in both gps (similar results in AN and BN)

		BDI score improved* in both gps, more in 1* (similar results in AN and BN)<br>↓ of BP episodes >75%:<br>At 8 weeks: n=48 (92%) vs n=22 (43%) *<br>At 3 months FU: n=46 (88%) vs n=23 (45%) *<br>At 12 months FU: n=43 (82%) vs n=23 (45%) *<br>Similar results in AN and BN.<br>OI improvement at 8 weeks higher* (similar results in AN and BN)<br>In AN subgps:<br>BP abstinence achieved at 8 weeks: 84% vs 35%*<br>BDI scores improvement and BP episode ↓ were linked*
Zuercher et al., 2003 [41]	1. NG (n=155)<br>2. OI (n=226)	1. vs 2.:<br>EDI-2 scores post treatment similar.<br>Treatment satisfaction levels similar

ACQ: anxiety control questionnaire - perceived control over anxiety; adm: admission; AN: anorexia nervosa; BAI: Beck Anxiety Inventory; BDI: Beck Depression Inventory; BMI: body mass index; BN: bulimia nervosa; BP: binge purge; CBT: cognitive behavioural therapy; d/c: discharge; % EBW: percentage expected body weight; ED: eating disorder; EDE-Q: Eating Disorder Examination Questionnaire; EDI: Eating Disorder Inventory; EDRC: Eating Disorder Risk Composite; FU: follow up; gp: group; %mBMI: percentage median BMI; mon.: month; MS: medical stabilisation; NG: nasogastric tube feeding group; NG-R: NG feeding under restraint; NR: not reported; n.s: not statistically different; OI: oral intake group; QoL: Quality of Life score;. STAI -Y1 and -Y2: Speilberger State and Trait Anxiety Inventory – Y1: trait anxiety. Y2: state anxiety; subgp: subgroup; WR: weight restoration; w/NG: with NG nutrition; * significant difference ** randomised controlled trials
