# Supplementary material for: Enteral Tube Nutrition in Anorexia Nervosa and Atypical Anorexia Nervosa and Outcomes: A Systematic Scoping Review
Source: Nutrients. 2025 Jan 24;17(3):425. doi: 10.3390/nu17030425 (PMC11820346; doi:10.3390/nu17030425)
Supplement: Supplementary file 1 [file nutrients-17-00425-s001.zip › Table S3 qualitative data table 17.01.25.pdf]

Table S3: Summary of qualitative data

Author, year	Setting	Characteristics of participants:<br>***Diagnosis (n)***<br>**Gender (%)**<br>*Age range*<br>Other:	Study question<br><br>***Nutritional provision***	Themes	Relevance
Fuller et al., 2023 [22]	United Kingdom, in the community	Lived experience participants:<br>***AN (n=7)***<br>**Female (100%)**<br>*19-54 yrs*<br>Experience of NG-R in the past – 14 mon to 30 yrs ago<br>.<br>Parents/ carers participants (n=13):<br>**Female (n=11, 85%)**<br>**male: (n=2, 15%)**<br>Loved one who had experienced NG-R when they were 12-27 yrs.<br><br>Clinicians (n=16):<br>**Female (n=11, 69%)**<br>**Male (n=5, 31%)**<br>Duration of experience in mental health: 10 months – 17 yrs	Evaluating impact of previous NG-R as part of treatment of AN. Impact on patients, carers and MDT in units where NG-R was administered.<br><br>***Nutrition: NG-R.***	1) Impact of NG-R on participants and others around them: trauma; intrusiveness; being surrounded; feeling 'hated'. New 'benchmark' reached; the need to continue NG-R; feeling stuck.<br>2) Isolation from others; strengthening alliance with AN. Peers not understanding them; 'in a bubble'.<br>3) Clinicians perceived breakdown of trust with patient.<br>4) Impact of seeing others being restrained – traumatised; haunted by screams; triggering; transported to when they experienced it.<br>5) Parents remembering trauma of hearing, potentially, their child scream; empathic towards staff needing to do a difficult job, may be exhausted and traumatised themselves.<br>6) Longer term impact: - PTSD symptoms – for example when witnessing an NG procedure in someone else, or during covid testing; diagnosis of PTSD.	- Lifesaving treatment<br>- However, awareness of impact/ long term negative impact<br>- Impact on people around the person being restrained<br>- To move away from 'alliance with AN + NG' towards integration with wider community<br>- Awareness of the possibility of repeating the same pattern

		Professional backgrounds: varied		7) Positive impact of NG: Difficult experience, but relating to recovery; grateful to have got a life, making peace with it. Clinicians encountering patients who were grateful.	
Fuller et al., 2024 [23]	United Kingdom, subjects in the community	As above	Evaluation of recommendations for best practice for NGR and how to reduce episodes of NGR<br><br>***Nutrition: NG R.***	1) Individualised care and how this may reduce trauma while encouraging common goal thinking.<br>2) Communication including a safe space for SUs and carers to be heard; reminders of why NG R is necessary; reminders of individual goals.<br>3) Importance of staff relationships with SUs. Treating SUs as human beings; kindness and compassion; building trusting relationships; fostering hope.	- Listening (clinicians) and feeling heard (LE and carers)<br>- Collaborative individualised plans – not just 'set protocols'<br>- Developing and fostering connections and hope for recovery even within the setting of difficult interactions such as NG-Rs.<br>- Actively looking to reduce trauma
Halse et al. 2005 [29]	Australia, adolescent medical unit	***AN (n=23 of which n=17 discussed NG experience)***<br>**Female (100%)**.<br>*12-20 yrs. Mean: 14.8 yrs.*<br>BMI mean: 15.6: range: 15.22-18.<br>Setting: inpatient	Finding meaning and perceptions of NG nutrition in AN<br><br>***Nutrition: NG alongside supported oral intake in 73% of patients***	1) Insertion of tube: Unpleasant; painful; conscious of the presence of a tube; worse experience when staff inexperienced with insertion or SU is anxious or resists insertion.<br>2) Necessary/ useful procedure: aid for recovery; medialised/scientific intervention; disguising nutrition; tricking AN.<br>3) Tube as a signifier of AN: to self and to others; may become part of 'self'.<br>4) Signifier of control: assertion of control between staff and patient; punitive especially if SU thinks they are trying	- Exploring meaning of NG in those needing it – and how to support transition away from NG<br>- Identity away from AN and the NG tube<br>- Thinking of 'finite' use of NG with regards to duration.

				their best; resistance to get control back, may be overt or covert.<br>5) Multiple/ contradictory meanings may be attached to the NG by same patient.	
Kezelman et al., 2016 [27]	Australia, adolescent medical unit	*AN (10)*<br>**Female (100%)**<br>*16-19 yrs. Mean ± s.d: 17.5 ± 0.79*<br>Admission BMI: mean ± s.d: 16.12 ± 1.53 kg/m^2<br>Setting: inpatient, rapid refeeding protocol	Evaluating psychological experience of inpatients on a rapid refeeding protocol<br><br>***Nutrition: NG 24 hr continuous 2400kcal/d initially. Continuing on NG until medically stable, then oral intake during day + nocturnal NG. Staged oral intake***	1) Reconciling with the diagnosis of AN: indicator of illness severity; challenging AN; need for clearer communication of targets and d/c targets emphasised.<br>2) Guilt of nutrition alleviated via NG.<br>3) Exposure to nutrition: lack of choice/ control; 'mechanical' eating, not challenging ED cognitions; no change in overall motivation; awareness of physiological sensations such as fullness overwhelming; increasing fullness through the day; these sensations continuing even with increasing ability to eat; meal supervision with interaction helpful; relating to other peers validating.<br>4) Consequences of weight gain: self-esteem concerns; worry of being back to weight where AN started; the term 'healthy' associated with less self-confidence; feeling that the team is focused on physiological rather than psychological well-being; lack of individuality; food aversion especially with oral intake and NG; those with a short duration of illness food enjoyment returning.	- Importance of severity recognition, alleviation of nutritional guilt in relation to NG – addressing this<br>- Acknowledging uncomfortableness of physiological sensations – psychological support to alleviate distress and anxiety<br>- Space for exploring feelings associated with NG and oral intake<br>- If guilt of eating alongside NG present – psychological support<br>- Clearer communication about expectations from SUs and in turn what to expect from team<br>- Exploring individualising care even if following a set protocol

Mac Donald et al., 2023 [28]	Denmark, subjects in the community	Lived experience:<br>***AN (n=7)***<br>**Female (n=7, 100%)**,<br>Lived experience of involuntary treatment (IT) >=5 events over at least 1 month, as part of AN management within the past 5 years, but not currently having IT.<br>n=4 (57%): current AN or AAN diagnosis.	Evaluating impact of multiple IT events in the context of AN treatment from lived experience perspective<br><br>***Nutrition: NG R.***	1) Living with internal coercion: AN dictated what they should or shouldn't do; ego-syntonic or partially ego-syntonic.<br>2) Coercive treatment: NG tube as a consequence of non-compliance; NG as a way of acceptance of nutrition without having to be responsible for eating; not having to taste or smell food; relief of nutrition being administered; reliance on tube feeding; IT accompanying NG nutrition; unpleasant, scary, inhumane when NG under restraint; mechanical restraint while feeding – degrading; post NG self-harm or purging resulting in more IT; NG while being mechanically restrained with acceptance or learned helplessness; some preferring mechanical restraint during NG as this will not 'give', but a human is likely to.<br>3) Leaving coercion: lasting imprint of fearing NG feeding and its associations including fear of touch & sensitivity to sounds; in retrospect, seeing the necessity for saving lives; motivated to avoid such experiences – being more caring of themselves.	- When SU felt 'as one' with AN, experience of IT with NG different when compared with how NG and IT were seen 'in retrospect' when they felt separate from AN.<br>- Consider how to improve SU connections with the team during treatment and less with AN<br>- When 'one with AN' – feeling coerced, resisting treatment, feeling the unpleasantness or self-punishment aspects of NG feed<br>- Encouraging self-care and human connections may support creating distance from punishment and from AN<br>- Looking back – SUs were able to see that staff were saving lives, that IT could not have been avoided, and yet had long-term effects in the way of trauma and wanting to avoid this experience from recurring<br>- To actively consider how to reduce trauma at the time of ETN

Matthews Rensch et. al., 2023 [21]	Australia, tertiary hospital medical unit	***AN/ AAN (n=8)*** **Female (100%)** *18-27 yrs (median 22 yrs)*. Setting: inpatient medical ward admitted for eating disorder treatment. NG for ≥ 7 days	Evaluating acceptability and experience of NG nutrition during refeeding. <br>***Nutrition: NG only, no oral intake for 1st 7 days.***	1) Expectations, awareness and involvement in treatment: feeling 'deceived', 'restricted', 'punished'; feeling shocked about NG only without oral intake, when previously managing some oral intake; feeling of lack of choice whether NG nutrition given or not. 2) Lack of communication: around treatment; questions unanswered; would have liked more information on NG use and rationale. 3) Treatment under MHA: dehumanising, depersonalising, 'a disorder', resentment about its use. 4) Pros and cons of NG only without food: Understood why NG but felt should have been only implemented if oral intake had failed; tampering of tube; uncomfortable/ traumatic/ reinsertion needed; anxiety about restarting food; lost the habit of eating regularly; comparing themselves with people who were given food; relief/ indifference; watching 'numbers' on pump constantly. 5) Interaction with others: positive or negative (competitive/ comparative). 6) Limited individualisation: proposed individual pathways.	- NG only either a relief or resented – space to express this - MHA and NG association – punitive, resentment, dehumanising – some way of communicating why needed to SUs + space for them to communicate their thoughts and feelings - SUs recommended individualised pathways - Worries over losing ability to eat – even with short term exclusive NG - SU - Recommended OI alongside NG

AAN: atypical anorexia nervosa; AN: anorexia nervosa; ED: eating disorder; LE: lived experience; MHA: mental health act; NG: nasogastric nutrition; NG R: NG under restraint; OI: oral intake; PTSD: post-traumatic stress disorder; SU: service user.
